# Connecting Mutations of the RNA Polymerase II C-Terminal Domain to Complex Phenotypic Changes Using Combined Gene Expression and Network Analyses

**DOI:** 10.1371/journal.pone.0011386

**Published:** 2010-06-30

**Authors:** Carlyle Rogers, Zhenhua Guo, John W. Stiller

**Affiliations:** 1 Department of Biology, East Carolina University, Greenville, North Carolina, United States of America; 2 Kunming Institute of Botany, Chinese Academy of Sciences (CAS), Kunming, China; Louisiana State University, United States of America

## Abstract

The C-terminal domain (CTD) of the largest subunit in DNA-dependent RNA polymerase II (RNAP II) is essential for mRNA synthesis and processing, through coordination of an astounding array of protein-protein interactions. Not surprisingly, CTD mutations can have complex, pleiotropic impacts on phenotype. For example, insertions of five alanine residues between CTD diheptads in yeast, which alter the CTD's overall tandem structure and physically separate core functional units, dramatically reduce growth rate and result in abnormally large cells that accumulate increased DNA content over time. Patterns by which specific CTD-protein interactions are disrupted by changes in CTD structure, as well as how downstream metabolic pathways are impacted, are difficult to target for direct experimental analyses. In an effort to connect an altered CTD to complex but quantifiable phenotypic changes, we applied network analyses of genes that are differentially expressed in our five alanine CTD mutant, combined with established genetic interactions from the *Saccharomyces cerevisiae* Genome Database (SGD). We were able to identify candidate genetic pathways, and several key genes, that could explain how this change in CTD structure leads to the specific phenotypic changes observed. These hypothetical networks identify links between CTD-associated proteins and mitotic function, control of cell cycle checkpoint mechanisms, and expression of cell wall and membrane components. Such results can help to direct future genetic and biochemical investigations that tie together the complex impacts of the CTD on global cellular metabolism.

## Introduction

The C-terminal domain (CTD) of RNA Polymerase II (RNAP II) comprises a sequence of tandemly repeated heptapeptides (Tyr_1_-Ser_2_-Pro_3_-Thr_4_-Ser_5_-Pro_6_-Ser_7_) that are essential for viability in both animals and yeast [1], [2]. The number of heptad repeats is relatively conserved within species but varies from yeast (26–28) to human (52) and across the animal, plant and fungal kingdoms [3], [4]. The CTD functions throughout the RNAP II transcription cycle by serving as a binding scaffold for a variety of protein-protein interactions involved in proper transcript initiation, elongation, and co-transcriptional mRNA processing [5]. It also participates in other diverse processes, including chromatin remodeling, DNA repair, and packaging, editing, and export of mRNAs from the nucleus [6]. Because it is so central to so many cellular processes, the CTD has been the focus of numerous genetic investigations, with a particular focus on how mRNA synthesis and processing are regulated [7].

The essential elements required for CTD function have been determined in yeast through substitution, deletion and insertion mutations [8], [9], [10]; the core CTD functional unit lies within tandem heptapeptides or “diheptads”. In addition, CTD mutants with progressively longer polyalanine insertions between diheptads show a continuous decline in growth rates, and the induction of conditional phenotypes. This has been demonstrated for insertions up to five Ala residues (5A) [9]; however, restoring the global amino acid register by extending insertions to seven alanines between diheptad units proved to be lethal, leading to the conclusion that too great a separation between functional units puts undue stress on at least some essential CTD-protein interactions [10].

**Figure 1:**
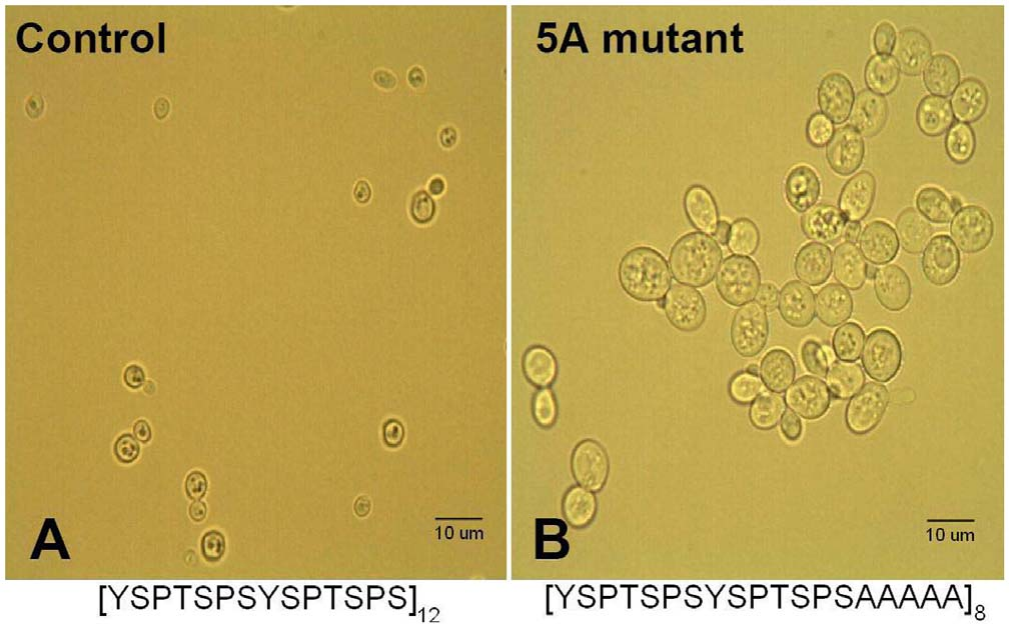
Yeast mutant phenotype. Representative 1000X photomicrographs of A) control cells containing the WT CTD and B) 5A Mutants after two rounds of exponential growth. The sequence of the tandemly repeated RNAP CTD present is shown below each respective cell line.

Through an ongoing investigation of functional constraints responsible for patterns of evolutionary conservation of the CTD [4], [9], [11], we have developed a number of yeast CTD mutants that exhibit various complex phenotypes. Most mutations of the CTD in yeast have pleiotropic effects on one or another major feature of cellular metabolism, including growth rate, cell size, budding pattern, capacity to adjust to physical or metabolic stress, and how efficiently the CTD is phosphorylated by CTD-directed kinases [1], [3], [8], [9]. Because its effects are transduced through largely uncharacterized pathways of protein-protein interactions, how a CTD mutation leads to a given suite of pleiotropic effects can be difficult to unravel. Therefore, we investigated one of our CTD mutants, which contains regular insertions of 5 alanines between CTD diheptapeptides, hereafter referred to as the “5A mutant” (see Fig. 1). 5A mutant cells exhibit both abnormal accumulation of excess DNA and larger cell size.

We explored potential genetic interactions that could contribute to these two specific phenotypes, using a combination of microarray data and network analyses of known genetic interactions in yeast. Our goal was to investigate the utility of network analyses of empirical data for understanding how structural changes in the RNAP II CTD are transduced through metabolic pathways to produce the complex phenotypes exhibited by many CTD mutants. We were able to anchor our networks with specific phenotypic changes on one end, which could be used to define functional categories of gene networks to analyze, and a single genetic change (5A insertion mutations of the CTD) on the other. The hypothetical networks we developed point to specific pathways that connect the CTD to various genes, a number of which have been shown previously to be implicated in similar phenotypic changes. Our results suggest that network analyses can be a useful tool for helping to understand how the CTD regulates broader cellular functions.

## Results

### Quantification of cell size differences in the 5A CTD mutant

In addition to the substantially reduced growth rates reported previously [9], we noticed that 5A mutant cells were abnormally large under microscopic observation. Initially, we quantified this difference using digital photomicroscopy. Control yeast cells (transformed with the WT CTD) had, on average, an image area of 12.34 µm^2^ (n = 82) when measured at log phase growth (Fig. 1A). In contrast, 5A mutants proved to be significantly larger, growing to an average apparent area of 24.28 µm (n = 76) (Fig. 1B) during the third round of growth (see methods) after initial transformation (p<0.0005 in a t-test against control cells). The average size of mutant cells increased over time to 32.43 µm (n = 79) at the completion of 20 rounds of growth, while size of control cells did not change. In addition, average log phase doubling time of 5A mutants was 18 hours in the first round of growth (see methods), compared to two hours for control cells. As 5A mutants were taken through multiple exponential growth cycles, however, doubling times increased by approximately two hours per cycle. There appeared to be no further increase once average doubling times of 5A mutant cultures reached 24 hours, and no change in doubling time was observed in control cells over time. Delays in completion of the yeast cell cycle [12] and the accumulation of large, abnormally budding cells [13] both have been linked to an increase in chromosome content or aneuploidy. Because these phenotypic differences are present in the 5A CTD mutant, we further explored the possibility of abnormal DNA content using flow cytometry.

### Measurement of DNA content through flow cytometry

**Figure 2:**
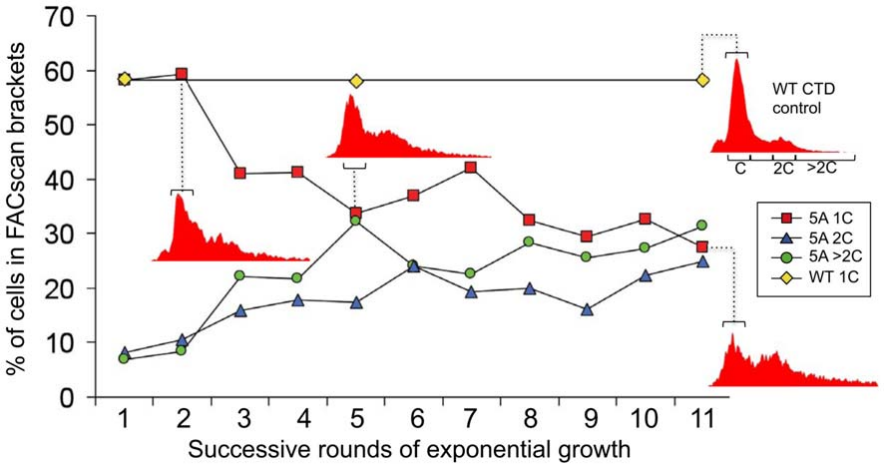
FACscan analysis. The percentages of cells (based on brackets shown on the WT control cell FACscan profile), with different levels of DNA content over a time course of growth cycles of 5A mutant versus the control strain containing the WT CTD (C = 1 chromatid per chromosome, or the normal yeast haploid DNA content in G1 phase). Flow cytometry histograms depicting the shift of DNA content at early to mid log phase over a series of growth cycles. Dotted lines connect the data point for 1C content to the bracket in the histogram recovered from cells in the respective growth cycle. Each cycle comprised an initial inoculation, followed by growth through log phase to stationarity.

Both mutant and control cells were fixed during log phase growth, stained with propidium iodide, and analyzed for DNA content using FACScan. The yeast cell cycle consists of two growth phases G1 and G2, interrupted by S phase in which chromosomes are replicated, and culminating in mitosis [14]. Based on our flow data, control cells spend, on average, 60% of their time in G1 during exponential growth (Fig. 2); that is, at mid-log phase time points sampled, 60% of cells counted fell under the 1C peak (size of normal haploid genome). 5A mutant cells measured during the first round of growth after transformation (see methods) appeared similar to the control cells, also with 60% of the cells under the typical 1C peak (Fig. 2).

Over the time course investigated, control cells showed no measurable changes in the profile of DNA content when harvested during mid-log phase growth. In contrast, although 5A mutant cultures began with a similar DNA profile to control cells, they showed a continuous decrease in the proportion of cells with 1C content over time, and an increase in cells with 2C and greater DNA content (Fig. 2). Under the assumption that mutant cells in log phase spend most of their time in the G1 phase of the cell cycle, this shift indicates that average DNA content has increased through time, and that many 5A mutant cells in G1 phase are counted under or beyond the 2C peak (Fig. 2).

An increase in the proportion of cells found under the 2C peak also would be consistent with cells delaying in G2 phase; however, the continuous increase of cells beyond the 2C content bracket indicates that many mutant cells are not simply spending proportionally longer in G2 phase, but are accumulating abnormally large amounts of DNA. Over the course of 11 growth cycles, the frequency of 5A mutant cells measured with 1C content declined, while those with 2C or greater increased continuously (Fig. 2). Along with a parallel decline in average growth rate, FACScan results suggest a growing proportion of aneuploid cells accumulate in 5A mutant cultures over time. Interestingly, extension of the time course to a 20th round of growth indicated no measurable change in average DNA content of cells in 5A mutant cultures compared to 11 rounds. Consistent with the observed stabilization of doubling time (see above), this suggests that cultures reach equilibrium between formation of viable aneuploid cells and mortality caused by genetic imbalances. It also is possible, however, that our flow data do not accurately reflect the number of cells at the larger end of the distribution (greater than 90 µm^2^), due to their disproportionate loss during washing and fixation, and/or preferential removal during FACScan gating.

### Analyses of gene networks based on microarray data

Using microarray analyses, we were able to identify 818 genes out of 6221 that were expressed differently in 5A mutant compared to control cells, based on 0.5 and 2 fold expression ratios as cut-off values. Among them, 325 genes/ORFs were up-regulated and 493 down-regulated (See file s1 for details on differentially expressed genes). These expression data have been deposited in the NCBI Gene Expression Omnibus [15] and are accessible through GEO Series accession number GSE14342 (http://www.ncbi.nlm.nih.gov/geo/query/acc.cgi?acc=GSE14342).

**Figure 3:**
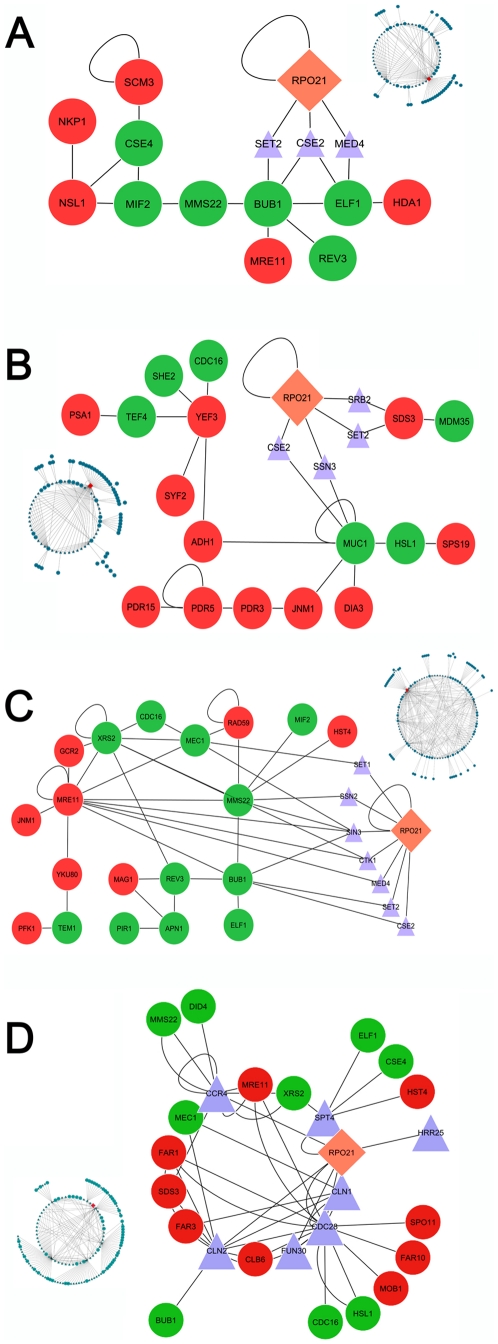
CTD and direct interaction networks. Combined networks linking first interactions of RPB1/RPO21 with direct interactions among differentially expressed genes. A) Chromosome segregation network, including proteins involved in functions such as chromosome segregation, mitotic segregation, kinetochore, and mitotic spindle assembly. B) Cell wall and membrane network, including genes related to sporulation, cell wall synthesis, cell wall structure, and plasma membrane components. C) Cell cycle and repair network, including different break repair strategies, cell cycle signals, and responses to DNA damage. D) A network expanding possible connections to direct RNAP II interactors that have not been demonstrated to interact specifically with the CTD. This network includes genes that are differentially expressed in the 5A mutant, and for which there is prior experimental evidence of similar phenotypic effects as those exhibited in 5A cells. In all panels, pink diamonds indicate RPB1/RPO21, blue triangles are first interactions of RPB1, and circles are differentially regulated genes. Red circles indicate down regulation, green indicate up regulation, and light blue indicates no change in regulation (restricted to sequences that interact directly with the CTD in final networks). Insets in each panel show the complete union of RPB1 plus direct interaction expression networks for that functional category, from which the CTD plus first interaction sub-networks were extracted. Larger, versions of the inset full networks are provided in figures S2, S3, S4, S5.

The *Saccharomyces* genome database (SGD) contained no functional annotation for 277 of these genes and, therefore, they were not included in network constructions. Because we could anchor networks on one end with a known CTD mutation, and on the other with defined phenotypes, we sought to build metabolic networks that could help to guide future research on the pathways in between. Based on 5A mutant phenotypes, we chose three categories to construct sub-networks of “direct interactions” among differentially regulated genes; (i) chromosome segregation, (ii) cell wall and membrane biosynthesis, (iii) cell cycle regulation and DNA repair (Fig. 3). Genes were included in networks if they were differentially regulated and also fell into one of the identified categories based on SGD annotation.

Although we based construction of our metabolic networks on observed mutant phenotypes, as a further validation of our GO categories we performed clustering with Cytoscape plug-in Bingo 2.3 [16]. This allowed us to examine whether differentially expressed genes in our predicted GO categories were significantly overrepresented in the microarray data. Using Bingo 2.3, we ran a hypergeometric test on all differentially regulated genes, with an output of overrepresented GO categories, using a Benjamini and Hochberg false discovery rate correction and a significance level of 0.05. This analysis showed significant enrichment in GO categories associated with cell wall metabolism, chromosome organization and biogenesis, and mitotic recombination. Although this result helps to validate our choices of metabolic categories for network analyses, it is important to note that gene functional annotations and, therefore, GO classifications currently are incomplete. Consequently, the lack of significant GO assignments in other metabolic categories does not necessarily indicate an absence of biological relevance. Rather, it can be viewed as an opportunity to discover novel biological function [17].

To create complete networks that can be tied specifically to CTD mutations, we also identified all proteins in the database that exhibit “first interactions” with the CTD, and determined where they intersect with “direct interactions” of differentially regulated genes from each of the phenotype sub-networks described above. It should be noted that CTD “first interaction” proteins could impact yeast phenotypes either because they cannot interact properly with the altered 5A CTD protein, or because they are, themselves, differentially expressed as a result of 5A mutations. Although based on our microarray data a number of proteins known to bind the CTD are encoded by genes differentially expressed in the 5A mutant, no CTD first interactors included in our networks turned out to display significantly altered expression (see figure S1 for details on CTD interactors).

In annotating genetic interactions, the SGD does not distinguish between the CTD and other regions of the RPB1/RPO21 subunit of RNAP II. Although yeast is exceptionally well studied, many interactions between the CTD and associated proteins remain uncharacterized [18], [19]. We took a conservative approach, only including proteins that either contain an annotated CTD binding domain, or are known to interact directly with the CTD through empirical research. Below we describe three functional gene expression networks that link CTD first interactions to observed phenotypic changes in 5A mutant cells.

### Chromosome Segregation Network

Mitotic cell division ensures that chromosomes are faithfully replicated and segregate equally between mother and daughter cells, as the absence or irregular numbers of genes is typically deleterious or lethal [20]. In many eukaryotes, chromosomal movements during mitosis are mediated by conserved mechanisms involving three structures: the bipolar mitotic spindle, kinetochores (centromere DNA and associated proteins), and the centrosomes (microtubule organizing centers) [20].

We identified a chromosome segregation network of genes involved in proper kinetochore function that also can be linked to proteins that interact with the RNAP II CTD (Fig. 3A). Most notably MIF2 and CSE4 show increased expression, while NSL1 and SCM3 are down-regulated in the 5A mutant. It has been shown previously that when MIF2 is over-expressed, chromosomes mis-segregate during mitosis and cells accumulate in the G2 and M phases of the cell cycle as large buds [21]. Mutations in CSE4 also result in large budded cells and an increase in the frequency of nondisjunction [22]. SCM3 is involved in the localization of CSE4, and SCM3 deletion mutants show disrupted localization of the centromere [23]. Finally, NSL1 is essential in yeast, and mutations lead to large budded cells and defects in microtubule formation [20]. Our network analyses implicate pathways through which regulation of these genes could be influenced by the mutated CTD, thereby contributing to large size, abnormal budding and possible aneuploidy in 5A mutant cells (Fig. 3A).

Cells rely on checkpoint surveillance mechanisms to ensure proper genome replication and promote high fidelity of the division cycle [12]. A key node in our chromosome segregation network analyses is occupied by the over-expressed BUB1 gene (Fig. 3A), which is part of a checkpoint that delays the onset of anaphase in cells with defects in mitotic spindle assembly, or in the attachment of chromosomes to the spindle microtubules. Research has shown that over expression of a dominant mutant, BUB1-5, can delay mitosis [24], which could help to explain the observed increase in doubling time in 5A cells. Additional up and down regulated genes in this network are shown in figure 3A.

### Cell Wall and Membrane Network

Although our expression data suggest many possible genes that could result in abnormal DNA content and, indirectly, in cell budding and size effects, we also were interested in examining possible gene pathways that could contribute directly to increased average size of 5A mutant cells. We therefore formed a network based on the intersection of CTD first interactions with a sub-network of differentially expressed genes known to be involved in cell wall and membrane synthesis, sporulation, and cell growth.

This network identified a number of genes that could contribute to increased cell size (Fig. 3B). Of particular interest are CDC16, HSL1, PSA1, and genes listed as ATP-binding cassettes. CDC16 is an essential member of the anaphase-promoting complex (APC) and several temperature-sensitive mutants CDC16 arrest as large-budded cells with the nucleus at the neck [25]. HSL1 mutants also exhibit abnormally elongated buds [26] and mutants of PSA1, which synthesizes GDP-mannose for incorporation into N-linked and O-linked glycoproteins, have defects in cell wall biosynthesis [27], [28].

This network also included a number of genes for ATP-binding cassettes (ABC) PDR15, PDR3, and PDR5, all of which play important roles in drug efflux and may also function in cellular detoxification [29]. The relevance of these genes is in their abilities to regulate other genes involved in DNA damage repair [30]. Problems with regulation of DNA damage repair and chromosome mis-segregation have been found in aneuploid cells with mutated cohesion proteins. These mutants display relevant reactions to internal and external stress stimuli, including changes in DNA damage repair, mitochondria function, and oxidative stress, all of which play important roles in yeast apoptotic cell death [31]. Additional genes in this network are shown in figure 3B.

### Cell Cycle and DNA Repair Network

Because we recovered BUB1 as a key node in the chromosome segregation network, we decided to investigate whether additional checkpoint or repair mechanisms could be identified in a network of differentially expressed genes in the 5A mutant (Fig. 3C). The networked genes involved in cell cycle checkpoints included PFK1, TEM1, HST4, SIN3 and, of particular interest, MEC1. Mutations in MEC1 have been shown to lead to multiple defects, including sensitivity to DNA damage, impaired checkpoint functions, chromosome breakage, and loss of telomeric silencing [32], [33].

In addition to checkpoint-related genes, this network also recovered APN1, MAG1, and REV3, which all are involved in DNA repair [34], [35], [36]. Interestingly, three genes (XRS2, RAD59, and YKU80) found in the network are implicated not only in DNA checkpoint controls, but also double-strand break repair using both homologous and nonhomologous mechanisms [37], [38], [39]. All genes in this network are shown in figure 3C.

## Discussion

Although microarray data allowed us to identify differentially expressed genes in functional categories related to observed 5A mutant phenotypes, and to build pathways among these genes, there is no indication that most products of these genes have direct interactions with the RNAP II CTD. Likewise, of the proteins known to interact with the CTD, none that show altered expression in 5A mutant cells are implicated in the phenotypic changes observed. In contrast, by connecting sub-networks of genes identified in microarray analyses, with proteins known to bind the CTD, we were able to form putative connections between phenotypic changes and the specific CTD alterations introduced. As discussed above (and more extensively in our methods section), CTD associated proteins may or may not be differentially expressed (those connected to our specific functional networks were not); however, in either case their downstream effects can be further modulated by reduced efficiency of their direct physical interactions with the mutated CTD. For example, like CSE2 discussed below, HRR25 is a protein kinase that contains a CTD binding domain and has been shown to be involved in regulating DNA repair and chromosome segregation [6], [40].

Because we were conservative in building our CTD “first interactions” sub-network, limiting it to proteins for which there is specific evidence of a CTD interaction, its intersections with gene expression networks are likely to be missing important nodes. This undoubtedly includes some genes that are differentially expressed in the 5A mutant, but could not be connected to a direct interaction with the CTD. There are many additional genes in the SGD that are annotated as interacting with the RNAP II largest subunit; however, binding domains in CTD associated proteins are not well conserved [10], making it difficult to assign a given RPB1 protein interaction to the CTD without experimental evidence. Nevertheless, given the remarkable number of CTD-protein interactions already established [6] there undoubtedly are additional networks of genes connected to the CTD that play a role in the complex phenotypes of our 5A mutant.

To investigate additional possible connections between 5A CTD mutations and phenotypic changes, we relaxed our requirement for a demonstrated CTD-protein interaction and created one additional interaction network (Fig. 3D). It linked differentially expressed genes with empirically demonstrated effects similar to 5A mutant phenotypes, to first interactors with RPB1 that have not been tied to the CTD experimentally. This network not only recovered genes in the chromosome segregation pathway discussed above (BUB1, ELF, CSE4), but also genes such as MOB1 that is required for mitotic exit, and CLB6 that is involved in mitotic spindle formation. Interestingly, FAR1, FAR3, FAR10, CLN1, CLN2, and CDC28 play roles in cell cycle regulation either by promoting its continuation or its arrest. An interesting aspect of this finding is the ability of CLN1 and CLN2 to form a complex with CDC28 to promote progression through the cell cycle [12]. Experimental evidence shows that prevention of CLN2 accumulation can cause cells to delay in G1 [41], [42], a characteristic of aneuploid cells [12] and one that is consistent with the reduced growth rate of 5A mutant cells. Additionally, PIN1, which is required for chromosome condensation, acts to stimulate hyperphosphorylation of the CTD, affecting transcription and the cell cycle [43], [44]. The CTD plus direct interaction networks associated with abnormal DNA content are even more complicated when examining the role of mediator.

### Mediator and CTD mutant phenotypes

The mediator complex is required for regulation of most RNAP II transcription [45]. It is composed of multiple subunits organized into three regions; the head, middle, and tail. Domains of the head and middle interact directly with the CTD, however, additional research is needed to elucidate exactly which subunits play a role in binding [46]. In the three functional interaction networks we created (Figs. 3A–C), three different mediator proteins are found (MED4, SRB2, and CSE2); of central interest is CSE2, found in the middle region [46]. CSE2 mutants experience chromosome mis-segregation, large budded cells, elongated yeast bodies, and slow growth [13]. In other words, known CSE2 mutant phenotypes are very similar to our 5A CTD mutant; combined with its appearance in all three networks, disruption of possible CSE2-CTD binding appears as a most promising explanation for why 5A insertions into the CTD lead to large cells with abnormal accumulations of excess DNA. Thus, our network analyses point to CSE2 as a potentially key node in CTD-transduced metabolic networks, and suggest new directions for experimental research into the specific mechanics of CSE2-CTD interactions.

Sequential changes in the phosphorylation state of the CTD order and orchestrate the roles of its various proteins partners throughout the transcription cycle [6]. Mediator is required not only for transcriptional suppression, but also for the stimulation of basal transcription and regulation of CTD phosphorylation efficiency [45], [46], [47]. *In vitro* experiments using CTD kinases CDK7/CycH/MAT1, CDK8/CycC (SSN3, found in the middle region of the mediator), CDK9/CycT1, and yeast CTDK-I, all showed a sharp decrease of *in vitro* phosphorylation of a 5A-mutated CTD fusion protein relative to a WT CTD control sequence [10]. Thus, altered phosphorylation could explain why CSE2 and/or other first interactions with the 5A mutated CTD are disrupted. This, in turn, would initiate downstream cascades of altered gene regulation, leading to defective chromosome replication or segregation, large cells, and slow growth. Certainly it is also possible that CSE2 and other specific associated proteins could have trouble binding the 5A mutated CTD, even were it properly phosphorylated [10]. Thus, empirical data on the structure of CTD docking domains in CSE2 would indicate whether the insertion mutations interfere directly with CTD binding, or whether the effects are indirect and due to changes in post-translational CTD modifications.

### Conclusions

Our novel CTD/gene/protein network analyses point to previously uncharacterized pathways important for maintaining proper genome maintenance and cell division in yeast. To our knowledge, examination of 5A CTD mutant phenotypes, and their underlying genetic bases, provides the first specific evidence for a role of the RNAP II CTD in several of these processes. The networks we constructed can be viewed as working hypotheses for how alterations of the CTD could be transduced to produce the pleiotropic effects observed. As highlighted above, the potential relevance of these pathways is supported by empirical studies of abnormal DNA content resulting from mutations of CTD associated proteins and of genes connected in our downstream direct interaction networks. One protein in particular, the mediator component CSE2, is identified as a key node connecting the CTD with a variety of the relevant genes with altered expression in 5A mutant cells. This conclusion finds strong empirical support from investigations with CSE2 mutants that exhibit very similar morphological phenotypes to 5A mutants [13]. Our results with CSE2, along with other genes highlighted, provide a foundation for further investigations into understanding the role of the CTD in maintaining genome integrity and controlling the cell cycle.

Given the number and complexity of CTD-protein interactions, zeroing in on the specific effects of different CTD mutations can be a daunting task. We have demonstrated that combining network analyses with empirical expression (microarray) data can provide novel insights into how the CTD could influence complex processes like genome duplication and cell wall formation. With a total of 818 genes out of 6221 genes showing significantly different expression in the 5A mutant, verification of the key interactions that cause observed phenotypes requires further experimental investigation. Nevertheless, our integrated approach shows promise for gaining insights into the role of the CTD in core processes in yeast, and for suggesting new mechanistic hypotheses that can be tested through direct empirical analyses.

## Methods

### Measurement of Cell Size

Yeast cells were transformed with mutated CTD sequences containing 5 alanine insertions between diheptad units via the plasmid shuffle, as described in greater detail previously [8], [9]. Briefly, complementary 5′-phosphorylated oligonucleotides were designed to encode the consensus CTD heptad in yeast, with additional Ala residues inserted between diheptads. Codon choices matched the most commonly used triplets in the yeast WT CTD. When annealed, the resulting double-stranded fragments were left with overhangs matching the two different *Ava*I recognition sites to facilitate directional cloning of concatenated fragments. Complementary oligonucleotides were annealed together and ligated into the pSBO vector. Because CTD truncation mutants with fewer than 13 repeats show at least conditional phenotypes, we screened artificial CTD sub-clones for inserts containing at least 13 WT heptapeptide motifs. The yeast WT CTD was replaced by mutated constructs via the plasmid shuffle. The yeast strain Z26 [48] was transformed by lithium-acetate treatment [49] and selected on synthetic complete (SC)-Leu-Ura medium to retain both the *URA3-*linked WT CTD and *LEU2*-linked mutated genes. Transformed colonies were replica-plated onto SC-Leu medium containing 5-fluoroorotic acid (5-FOA) to select cells without the *URA3*-linked RPB1^+^ gene [50].

Axenic transformed yeast cultures were grown to an optical density of 0.2 at 600 nm (OD600) in 10 mL of YPD (1% yeast extract, 2% peptone, and 2% dextrose) medium. Cells were placed on glass slides and photographed at 1000X under oil immersion. Cell size was determined using Motic Camera Plus software that integrates the observed area under a traced object. For unbiased sampling, slides were moved haphazardly through 15 fields of view, beginning with the objective at the edge of the cover slip. Pictures were taken of each new field of view until the opposite end of the cover slip was reached. All cells within a given frame were measured (average of six per frame). If budding or clumping was observed the largest cell in the group always was measured; if there were more than three cells in a clump, measurements were not taken.

### Flow Cytometry

Axenic yeast cultures were grown to an OD600 of between 0.2–0.5 in 10 mL of YPD, indicating early to mid log phase. Vortexing was implemented throughout the protocol to help break clumps, because sonication proved to be too harsh to permit consistent recovery of signal from 5A mutants. Approximately 1×10^6^ cells were harvested, pelleted in a tabletop centrifuge, and resuspended in 1 mL of 70% ethanol for 2 hours. Cells were washed twice in 2 mL of 0.05 M sodium acetate, resuspended in 1 mL of 0.05 M sodium acetate and 20 µL of 10 mg/mL of RNAase, incubated for 1 hr at 50°C, then for an additional hour with the addition of 35 µL of 10 mg/mL of proteinase K. Cells were washed and resuspended in 0.05 M sodium citrate and 35 µL of propidium iodide and analyzed for DNA content using a Becton Dickinson FACScan instrument. Flow cytometry outputs shown on dot plots were gated to exclude doublet signals. Gating was kept consistent throughout all samples to be conservative. A small 2C peak was visible in histogram plots in some FACS runs but not in others; however, percentages of cells under each predetermined marker range was similar in all measurements of control cells. Histograms of fluorescent signal, dot plots, and data statistics were analyzed in WinMDI 2.8 [51]. Flow cytometry markers were determined using the following equations, and the percentages of cells under each marker were calculated as follows, where *SSP = *Strongest G1 Signal Peak, *MR = *Marker Range (*SSP*/4): *G1* = *SSP* +/− *MR*, *G2* = 2 X *SSP* +/− MR, S-phase = interval between *G1* and *G2* brackets.

The time course for this experiment involved growing cells repeatedly to stationary phase and then inoculating fresh cultures. Measurements of cell size and DNA content were of cells that had been selected on 5-FOA plates, then transferred to YPD medium before clones were picked to inoculate the initial growth cultures examined. Thus, these mutant cells already had experienced an undetermined number of cell divisions before the start of our time course. After the freshly inoculated cultures had grown to early/mid log phase, a portion of the cells were fixed and analyzed as described above; the rest grew to stationarity and the process was repeated through 11 complete cycles. One additional measurement was taken after 20 of these growth cycles.

### Microarray analyses

RNA was extracted from fresh pellets of yeast cultures at log phase, grown to an OD600 of 0.8 in 100 ml of YPD medium at 30°C in a shaker at 225 rpm. Total RNA was extracted using Qiagen's RNeasy Kit (Valencia, CA). Four replicates for each sample yeast strain (5A and wild-type CTD control) were prepared, and 10 µg total RNA for each replicate was analyzed at the Duke University DNA Microarray Center. Array ID YO06N from Operon Yeast Genome Oligo Set version 1.1.2 was used for the hybridization. The direct labeling protocol was performed for sample RNAs, which included steps of first strand synthesis, slide preparation, hydrolysis, cDNA purification, hybridization and array washing. Cy3 and Cy5 were used for labeling the samples. Maui hybridization and TIGR washing system were used in this protocol for array hybridization and washing respectively. The full protocol can be found at http://www.genome.duke.edu/cores/microarray/services/spotted-arrays/protocols/. Fluorescent DNA bound to the microarray was detected with a GenePix 4000B scanner (Axon Instruments, Foster city, CA), using the GenePix 4000 software package to locate signal from spots. Normalization and statistical analysis were performed using Duke University BASE web server (https://base-server.duhs.duke.edu/). GO annotation was used for gene ontology. These expression data are MIAME compliant, and have been deposited in NCBI's Gene Expression Omnibus [15] and are accessible through GEO Series accession number GSE14342 (http://www.ncbi.nlm.nih.gov/geo/query/acc.cgi?acc=GSE14342).

### Network analyses

All differentially expressed genes in microarray analyses, along with known RNAP II-protein interactions downloaded from Thebiogrid.org, were loaded into the Cytoscape program (www.cytoscape.org; [52], [53]). GO categories were chosen based on clearly observed phenotypic changes. All differentially expressed genes from microarrays falling into specific categories designated in the Yeast Genome Database (YGD), e.g., “chromosome segregation,” “cell wall synthesis,” were selected to build genetic networks. The immediate or “direct interactions” of the selected genes were downloaded from www.thebiogrid.org and loaded into Cytoscape. Each group of direct interactions was designated as a network. Networks were merged and genes that were not connected to first interactions with RNAP II, or were not expressed differentially based on microarray results, were deleted. From the union of direct and RNAP II first interaction genes that remained, we selected only those that connected to RPB1 through a characterized CTD binding domain, or based on empirical evidence of a direct interaction with the CTD. As a final pruning step, only contiguous links between genes differentially expressed in microarray analyses and CTD-protein interactions were retained for further analyses of subset genetic networks.

## Supporting Information

Figure S1 All first interactions with the CTD of RNAP II. Genes indicated in purple are those that connect expression networks to the CTD, none of these genes show significantly altered expression in the 5A mutant. CTD interactors that are more highly expressed, but do not connect to one of our metabolic networks, are in green and those with lower expression in red. Genes shown in smaller, cyan triangles, neither connect to a network, nor show significantly altered expression.
(1.14 MB TIF)

Figure S2 Large version of Chromosomal segregation network
(10.16 MB TIF)

Figure S3 Large version of cell wall and membrane network
(9.10 MB TIF)

Figure S4 Large version of cell cycle and repair network
(1.53 MB TIF)

Figure S5 Larger version of possible connections to the CTD
(1.57 MB TIF)

File S1 Detailed description of all genes used in network analysis.
(0.19 MB PDF)
