# Supplementary figures and images for: Connecting Mutations of the RNA Polymerase II C-Terminal Domain to Complex Phenotypic Changes Using Combined Gene Expression and Network Analyses
Source: PLoS One. 2010 Jun 30;5(6):e11386. doi: 10.1371/journal.pone.0011386 (PMC2894937; doi:10.1371/journal.pone.0011386)

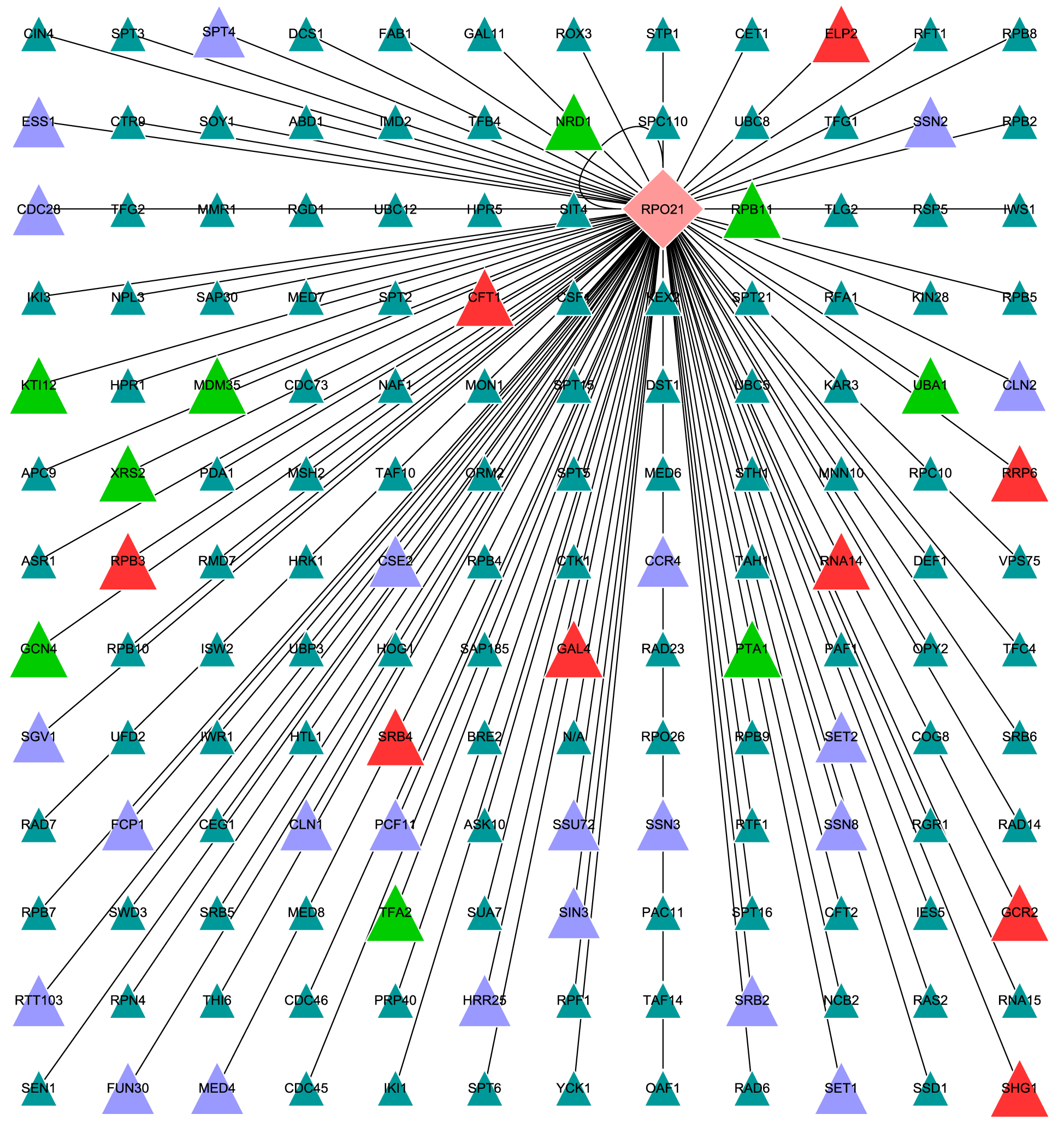

Supplement: Figure S1 — All first interactions with the CTD of RNAP II. Genes indicated in purple are those that connect expression networks to the CTD, none of these genes show significantly altered expression in the 5A mutant. CTD interactors that are more highly expressed, but do not connect to one of our metabolic networks, are in green and those with lower expression in red. Genes shown in smaller, cyan triangles, neither connect to a network, nor show significantly altered expression. (1.14 MB TIF) [file pone.0011386.s001.tif]

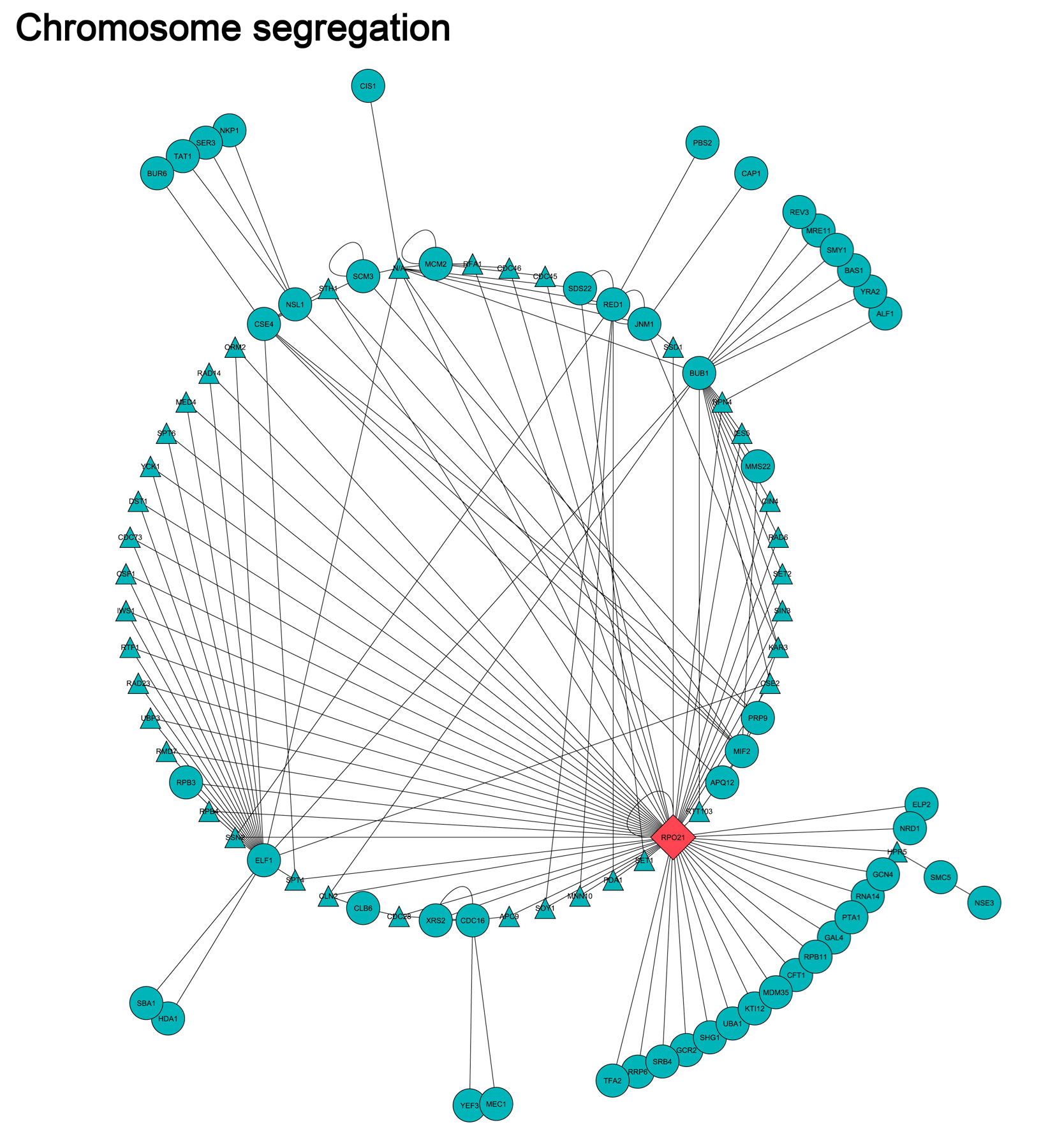

Supplement: Figure S2 — Large version of Chromosomal segregation network (10.16 MB TIF) [file pone.0011386.s002.tif]

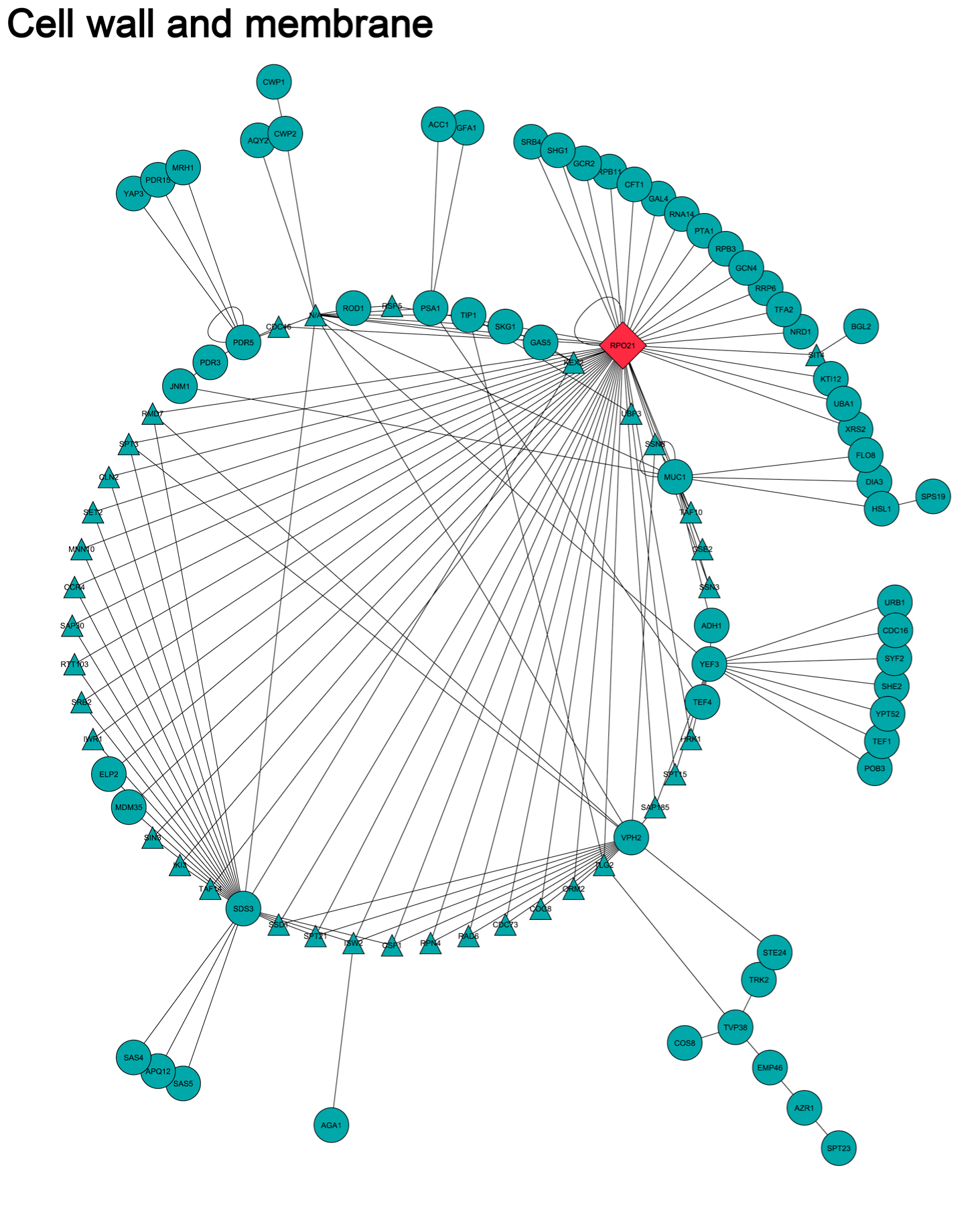

Supplement: Figure S3 — Large version of cell wall and membrane network (9.10 MB TIF) [file pone.0011386.s003.tif]

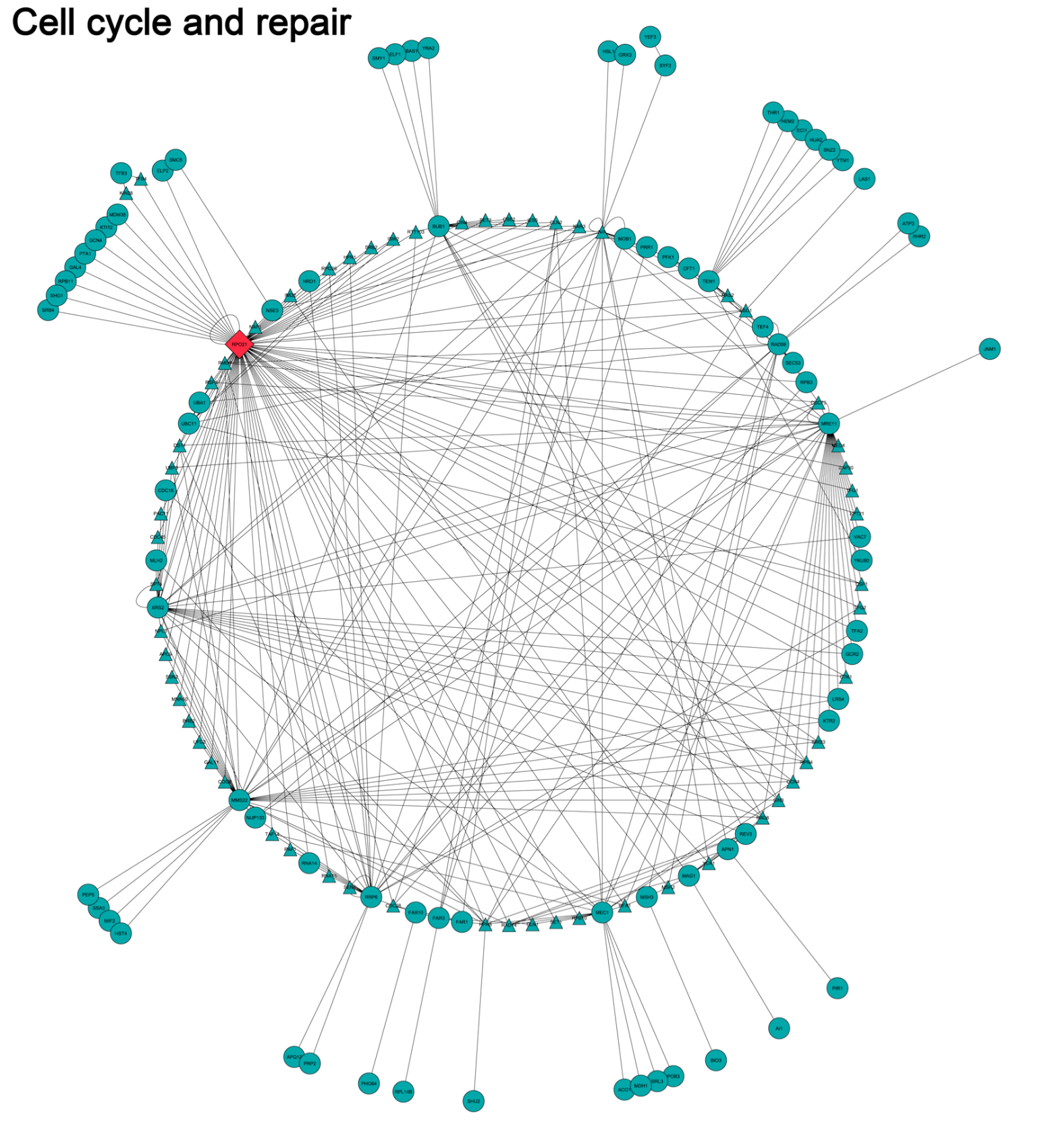

Supplement: Figure S4 — Large version of cell cycle and repair network (1.53 MB TIF) [file pone.0011386.s004.tif]

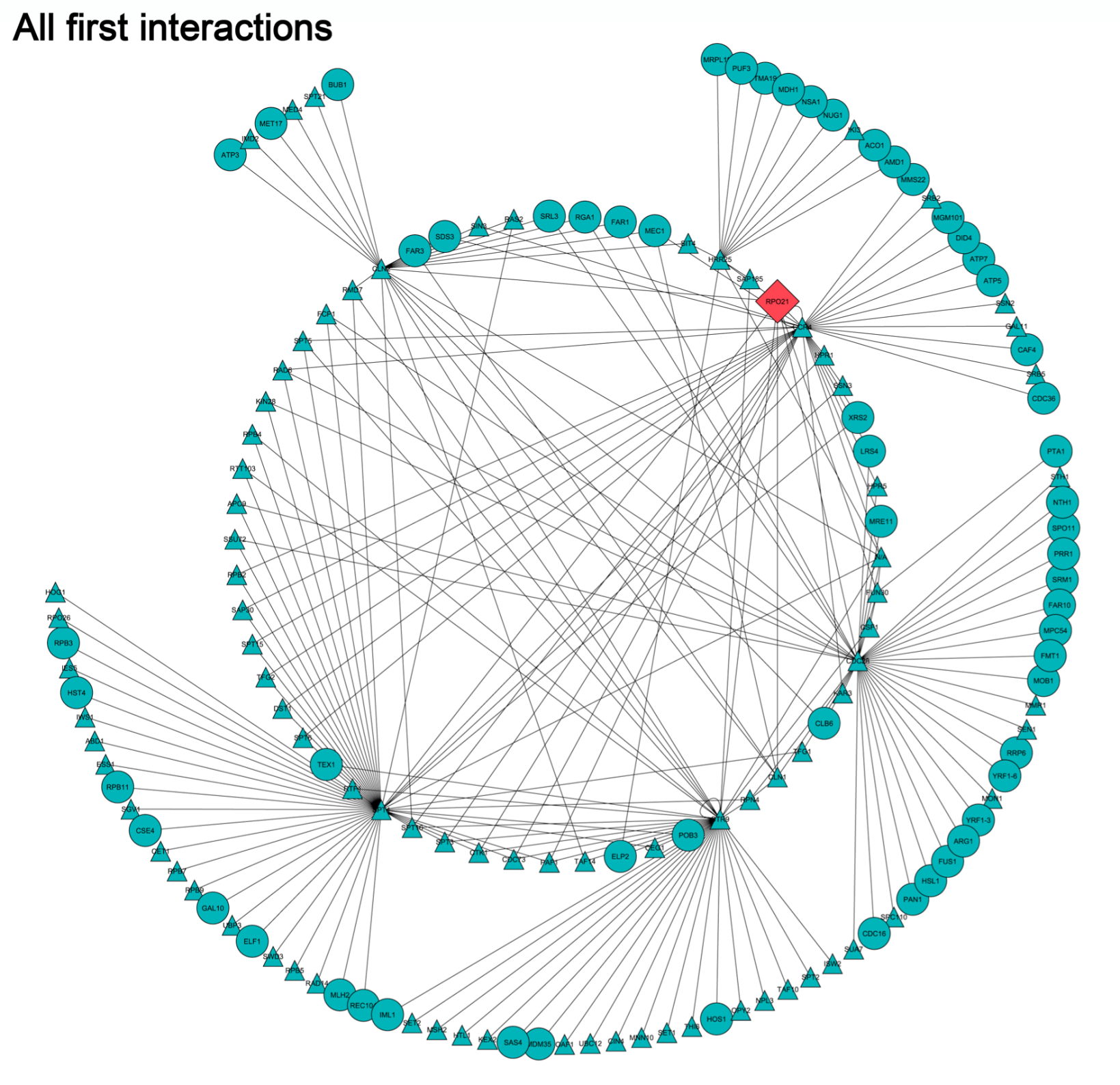

Supplement: Figure S5 — Larger version of possible connections to the CTD (1.57 MB TIF) [file pone.0011386.s005.tif]
