# Supplementary material for: Connecting Mutations of the RNA Polymerase II C-Terminal Domain to Complex Phenotypic Changes Using Combined Gene Expression and Network Analyses
Source: PLoS One. 2010 Jun 30;5(6):e11386. doi: 10.1371/journal.pone.0011386 (PMC2894937; doi:10.1371/journal.pone.0011386)
Supplement: File S1 — Detailed description of all genes used in network analysis. (0.19 MB PDF) [file pone.0011386.s006.pdf]

Gene Name	Alternate Gene Name	ORF	Up + or Down - regulation	Gene Description
AAD14		YNL331	-	Putative aryl-alcohol dehydrogenase with similarity to P. chrysosporium aryl-alcohol dehydrogenase; mutational analysis has not yet revealed a physiological role
AAD15		YOL165	-	Putative aryl-alcohol dehydrogenase with similarity to P. chrysosporium aryl-alcohol dehydrogenase; mutational analysis has not yet revealed a physiological role
AAD3		YCR107	-	Putative aryl-alcohol dehydrogenase with similarity to P. chrysosporium aryl-alcohol dehydrogenase; mutational analysis has not yet revealed a physiological role
AAH1		YNL141	-	Adenine deaminase (adenine aminohydrolase), involved in purine salvage and nitrogen catabolism
AAT1		YKL106	+	Mitochondrial aspartate aminotransferase, catalyzes the conversion of oxaloacetate to aspartate in aspartate and asparagine biosynthesis
ACC1	ABP2|FAS3|MTR7	YNR016	-	Acetyl-CoA carboxylase, biotin containing enzyme that catalyzes the carboxylation of acetyl-CoA to form malonyl-CoA; required for de novo biosynthesis of long-chain fatty acids
ACO1	GLU1	YLR304	-	Aconitase, required for the tricarboxylic acid (TCA) cycle and also independently required for mitochondrial genome maintenance; phosphorylated; component of the mitochondrial nucleoid; mutation leads to glutamate auxotrophy
ACP1		YKL192	+	Mitochondrial matrix acyl carrier protein, involved in biosynthesis of octanoate, which is a precursor to lipoic acid; activated by phosphopantetheinylation catalyzed by Ppt2p
ADD37		YMR184	+	Protein of unknown function involved in ER-associated protein degradation; green fluorescent protein (GFP)-fusion protein localizes to the cytoplasm and is induced in response to the DNA-damaging agent MMS; YMR184W is not an essential gene
ADD66	PBA2|POC2	YKL206	+	Protein involved in 20S proteasome assembly; forms a heterodimer with Pba1p that binds to proteasome precursors; similar to human PAC2 constituent of the PAC1-PAC2 complex involved in proteasome assembly
ADE17		YMR120	-	Enzyme of 'de novo' purine biosynthesis containing both 5-aminoimidazole-4-carboxamide ribonucleotide transformylase and inosine monophosphate cyclohydrolase activities, isozyme of Ade16p; ade16 ade17 mutants require adenine and histidine
ADH1	ADC1	YOL086	-	Alcohol dehydrogenase, fermentative isozyme active as homo- or heterotetramers; required for the reduction of acetaldehyde to ethanol, the last step in the glycolytic pathway
ADH2	ADR2	YMR303	-	Glucose-repressible alcohol dehydrogenase II, catalyzes the conversion of ethanol to acetaldehyde; involved in the production of certain carboxylate esters; regulated by ADR1
ADH5		YBR145	-	Alcohol dehydrogenase isoenzyme V; involved in ethanol production
ADY4		YLR227	-	Structural component of the meiotic outer plaque, which is a membrane-organizing center that assembles on the cytoplasmic face of the spindle pole body during meiosis II and triggers the formation of the prospore membrane
AGA1		YNR044	-	Anchorage subunit of a-agglutinin of a-cells, highly O-glycosylated protein with N-terminal secretion signal and C-terminal signal for addition of GPI anchor to cell wall, linked to adhesion subunit Aga2p via two disulfide bonds
AI1		Q0050	+	Reverse transcriptase required for splicing of the COX1 pre-mRNA, encoded by a mobile group II intron within the mitochondrial COX1 gene

Gene Name	Alternate Gene Name	ORF	Up + or Down - regulation	Gene Description
AI2		Q0055	+	Reverse transcriptase required for splicing of the COX1 pre-mRNA, encoded by a mobile group II intron within the mitochondrial COX1 gene
AI3		Q0060	+	Endonuclease I-SceIII, encoded by a mobile group I intron within the mitochondrial COX1 gene
AI4		Q0065	+	Endonuclease I-SceII, encoded by a mobile group I intron within the mitochondrial COX1 gene; intron is normally spliced by the BI4p maturase but AI4p can mutate to acquire the same maturase activity
AI5_ALPHA		Q0070	+	Endonuclease I-SceIV, involved in intron mobility; encoded by a mobile group I intron within the mitochondrial COX1 gene
AI5_BETA		Q0075	+	Protein of unknown function, encoded within an intron of the mitochondrial COX1 gene; translational initiation codon is predicted to be ATA rather than ATG
AIF1	CPD1	YNR074	-	Mitochondrial cell death effector that translocates to the nucleus in response to apoptotic stimuli, homolog of mammalian Apoptosis-Inducing Factor, putative reductase
AIM17	FMP12	YHL021	-	Putative protein of unknown function; the authentic, non-tagged protein is detected in highly purified mitochondria in high-throughput studies; null mutant displays decreased frequency of mitochondrial genome loss (petite formation)
AIM26		YKL037	+	Putative protein of unknown function; null mutant is viable and displays increased frequency of mitochondrial genome loss (petite formation)
AIM29		YKR074	+	Putative protein of unknown function; epitope-tagged protein localizes to the cytoplasm; null mutant displays increased frequency of mitochondrial genome loss (petite formation)
ALD6	ALD1	YPL061	-	Cytosolic aldehyde dehydrogenase, activated by Mg2+ and utilizes NADP+ as the preferred coenzyme; required for conversion of acetaldehyde to acetate; constitutively expressed; locates to the mitochondrial outer surface upon oxidative stress
ALF1		YNL148	-	Alpha-tubulin folding protein, similar to mammalian cofactor B; Alf1p-GFP localizes to cytoplasmic microtubules; required for the folding of alpha-tubulin and may play an additional role in microtubule maintenance
ALG3	RHK1	YBL082	+	Dolichol-P-Man dependent alpha(1-3) mannosyltransferase, involved in the synthesis of dolichol-linked oligosaccharide donor for N-linked glycosylation of proteins
ALY1		YKR021	+	Putative protein of unknown function; green fluorescent protein (GFP)-fusion protein localizes to the cytoplasm
AMD1	AMD3	YML035	-	AMP deaminase, tetrameric enzyme that catalyzes the deamination of AMP to form IMP and ammonia; may be involved in regulation of intracellular adenine nucleotide pools
APM3	YKS6	YBR288	+	Mu3-like subunit of the clathrin associated protein complex (AP-3); functions in transport of alkaline phosphatase to the vacuole via the alternate pathway
APN1		YKL114	+	Major apurinic/apyrimidinic endonuclease, 3'-repair diesterase involved in repair of DNA damage by oxidation and alkylating agents; also functions as a 3'-5' exonuclease to repair 7,8-dihydro-8-oxodeoxyguanosine
APQ12		YIL040	+	Protein involved in nucleocytoplasmic transport of mRNA

Gene Name	Alternate Gene Name	ORF	Up + or Down - regulation	Gene Description
AQY2		YLL052	+	Water channel that mediates the transport of water across cell membranes, only expressed in proliferating cells, controlled by osmotic signals, may be involved in freeze tolerance; disrupted by a stop codon in many S. cerevisiae strains
ARA2		YMR041	-	NAD-dependent arabinose dehydrogenase, involved in biosynthesis of erythroascorbic acid; similar to plant L-galactose dehydrogenase
ARG1	ARG10	YOL058	+	Arginosuccinate synthetase, catalyzes the formation of L-argininosuccinate from citrulline and L-aspartate in the arginine biosynthesis pathway; potential Cdc28p substrate
ARG3		YJL088	+	Ornithine carbamoyltransferase (carbamoylphosphate:L-ornithine carbamoyltransferase), catalyzes the sixth step in the biosynthesis of the arginine precursor ornithine
ARH1		YDR377	+	Oxidoreductase of the mitochondrial inner membrane, involved in cytoplasmic and mitochondrial iron homeostasis and required for activity of Fe-S cluster-containing enzymes; one of the few mitochondrial proteins essential for viability
ARN2	TAF1	YHL047	-	Transporter, member of the ARN family of transporters that specifically recognize siderophore-iron chelates; responsible for uptake of iron bound to the siderophore triacetylfusarinine C
ARR1	ACR1|YAP 8	YPR199	-	Transcriptional activator of the bZIP family, required for transcription of genes involved in resistance to arsenic compounds
ARR2	ACR2	YPR200	-	Arsenate reductase required for arsenate resistance; converts arsenate to arsenite which can then be exported from cells by Arr3p
ARR3	ACR3	YPR201	-	Arsenite transporter of the plasma membrane, required for resistance to arsenic compounds; transcription is activated by Arr1p in the presence of arsenite
ASP3-2	ASP3	YLR157	-	Cell-wall L-asparaginase II, involved in asparagine catabolism; expression is induced during nitrogen starvation; four copies of ASP3 are present in the genome reference strain S288C
ATG22	AUT4	YCL038	+	Protein required for the breakdown of autophagic vesicles in the vacuole during autophagy, putative integral membrane protein that localizes to vacuolar membranes and punctate structures attached to the vacuole
ATO3		YDR384	-	Plasma membrane protein, regulation pattern suggests a possible role in export of ammonia from the cell; phosphorylated in mitochondria; member of the TC 9.B.33 YaaH family of putative transporters
ATP15	ATPEPSIL ON	YPL271	+	Epsilon subunit of the F1 sector of mitochondrial F1F0 ATP synthase, which is a large, evolutionarily conserved enzyme complex required for ATP synthesis; phosphorylated
ATP17		YDR380	+	Subunit f of the F0 sector of mitochondrial F1F0 ATP synthase, which is a large, evolutionarily conserved enzyme complex required for ATP synthesis
ATP19		YOL077	+	Subunit k of the mitochondrial F1F0 ATP synthase, which is a large enzyme complex required for ATP synthesis; associated only with the dimeric form of ATP synthase
ATP20		YPR020	+	Subunit g of the mitochondrial F1F0 ATP synthase; reversibly phosphorylated on two residues; unphosphorylated form is required for dimerization of the ATP synthase complex
ATP3		YBR039	+	Gamma subunit of the F1 sector of mitochondrial F1F0 ATP synthase, which is a large, evolutionarily conserved enzyme complex required for ATP synthesis

Gene Name	Alternate Gene Name	ORF	Up + or Down - regulation	Gene Description
ATP5	OSC1	YDR298	+	Subunit 5 of the stator stalk of mitochondrial F1F0 ATP synthase, which is an evolutionarily conserved enzyme complex required for ATP synthesis; homologous to bovine subunit OSCP (oligomycin sensitivity-conferring protein); phosphorylated
ATP6	OLI2|OLI4|PHO1	Q0085	+	Mitochondrially encoded subunit 6 of the F0 sector of mitochondrial F1F0 ATP synthase, which is a large, evolutionarily conserved enzyme complex required for ATP synthesis
ATP7		YKL016	+	Subunit d of the stator stalk of mitochondrial F1F0 ATP synthase, which is a large, evolutionarily conserved enzyme complex required for ATP synthesis
ATP8	AAP1	Q0080	+	Subunit 8 of the F0 sector of mitochondrial inner membrane F1-F0 ATP synthase, encoded on the mitochondrial genome
AVT7		YIL088	-	Putative transporter, member of a family of seven S. cerevisiae genes (AVT1-7) related to vesicular GABA-glycine transporters
AYT1		YLL063	-	Acetyltransferase; catalyzes trichothecene 3-O-acetylation, suggesting a possible role in trichothecene biosynthesis
AZR1		YGR224	+	Plasma membrane transporter of the major facilitator superfamily, involved in resistance to azole drugs such as ketoconazole and fluconazole
BAR1	SST1	YIL015	-	Aspartyl protease secreted into the periplasmic space of mating type a cells, helps cells find mating partners, cleaves and inactivates alpha factor allowing cells to recover from alpha-factor-induced cell cycle arrest
BAS1		YKR099	+	Myb-related transcription factor involved in regulating basal and induced expression of genes of the purine and histidine biosynthesis pathways; also involved in regulation of meiotic recombination at specific genes
BCH2	FMP50	YKR027	+	Member of the ChAPs family of proteins (Chs5p-Arf1p-binding proteins: Bch1p, Bch2p, Bud7p, Chs6p), that forms the exomer complex with Chs5p to mediate export of specific cargo proteins, including Chs3p, from the Golgi to the plasma membrane
BDH2		YAL061	-	Zeta subunit of the oligosaccharyltransferase complex of the ER lumen, which catalyzes asparagine-linked glycosylation of newly synthesized proteins
BDS1		YOL164	-	Bacterially-derived sulfatase required for use of alkyl- and aryl-sulfates as sulfur sources
BET3		YKR068	+	Hydrophilic protein that acts in conjunction with SNARE proteins in targeting and fusion of ER to Golgi transport vesicles; component of the TRAPP (transport protein particle) complex
BGL2		YGR282	+	Endo-beta-1,3-glucanase, major protein of the cell wall, involved in cell wall maintenance
BI2		Q0110	-	Mitochondrial mRNA maturase with a role in splicing, encoded by both exon and intron sequences of partially processed COB mRNA
BI3		Q0115	-	Mitochondrial mRNA maturase, forms a complex with Mrs1p to mediate splicing of the bI3 intron of the COB gene; encoded by both exon and intron sequences of partially processed COB mRNA
BIO3		YNR058	+	7,8-diamino-pelargonic acid aminotransferase (DAPA), catalyzes the second step in the biotin biosynthesis pathway; BIO3 is in a cluster of 3 genes (BIO3, BIO4, and BIO5) that mediate biotin synthesis

Gene Name	Alternate Gene Name	ORF	Up + or Down - regulation	Gene Description
BIO5		YNR056	+	Putative transmembrane protein involved in the biotin biosynthesis pathway; responsible for uptake of 7-keto 8-aminopelargonic acid; BIO5 is in a cluster of 3 genes (BIO3, BIO4, and BIO5) that mediate biotin synthesis
BNA1	HAD1	YJR025	-	3-hydroxyanthranilic acid dioxygenase, required for biosynthesis of nicotinic acid from tryptophan via kynurenine pathway
BNA4		YBL098	-	Kynurenine 3-mono oxygenase, required for biosynthesis of nicotinic acid from tryptophan via kynurenine pathway
BNA5		YLR231	-	Kynureninase, required for biosynthesis of nicotinic acid from tryptophan via kynurenine pathway
BNA6	QPT1	YFR047	-	Quinolinate phosphoribosyl transferase, required for biosynthesis of nicotinic acid from tryptophan via kynurenine pathway
BOP2		YLR267	-	Protein of unknown function, overproduction suppresses a pam1 slv3 double null mutation
BOR1		YNL275	-	Boron efflux transporter of the plasma membrane; binds HCO3-, I-, Br-, NO3- and Cl-; has similarity to the characterized boron efflux transporter A. thaliana BOR1
BSC3		YLR465	-	Dubious open reading frame, unlikely to encode a protein; not conserved in closely related Saccharomyces species; 100% of YLR465C overlaps the uncharacterized ORF YLR464W and 86% of YLR465C overlaps the verified gene YRF1-4
BSC5		YNR069	-	Protein of unknown function, ORF exhibits genomic organization compatible with a translational readthrough-dependent mode of expression
BSC6		YOL137	+	Protein of unknown function containing 8 putative transmembrane seqments; ORF exhibits genomic organization compatible with a translational readthrough-dependent mode of expression
BUB1		YGR188	+	Protein kinase that forms a complex with Mad1p and Bub3p that is crucial in the checkpoint mechanism required to prevent cell cycle progression into anaphase in the presence of spindle damage, associates with centromere DNA via Skp1p
BUD19		YJL188	+	Dubious open reading frame, unlikely to encode a protein; not conserved in closely related Saccharomyces species; 88% of ORF overlaps the verified gene RPL39; diploid mutant displays a weak budding pattern phenotype in a systematic assay
BUD26		YDR241	+	Dubious open reading frame, unlikely to encode a protein; not conserved in closely related Saccharomyces species; 1% of ORF overlaps the verified gene SNU56; diploid mutant displays a weak budding pattern phenotype in a systematic assay
BUR6	NCB1	YER159	-	Subunit of a heterodimeric NC2 transcription regulator complex with Ncb2p; complex binds to TBP and can repress transcription by preventing preinitiation complex assembly or stimulate activated transcription; homologous to human NC2alpha
CAF17	IBA57	YJR122	-	Mitochondrial matrix protein involved in the incorporation of iron-sulfur clusters into mitochondrial aconitase-type proteins; activates the radical-SAM family members Bio2p and Lip5p; interacts with Ccr4p in the two-hybrid system
CAF20	CAF2|CAP 20	YOR276	+	Phosphoprotein of the mRNA cap-binding complex involved in translational control, repressor of cap-dependent translation initiation, competes with eIF4G for binding to eIF4E

Gene Name	Alternate Gene Name	ORF	Up + or Down - regulation	Gene Description
CAF4		YKR036	+	WD40 repeat-containing protein associated with the CCR4-NOT complex, interacts in a Ccr4p-dependent manner with Ssn2p; also interacts with Fis1p, Mdv1p and Dnm1p and plays a role in mitochondrial fission
CAP1		YKL007	-	Alpha subunit of the capping protein (CP) heterodimer (Cap1p and Cap2p) which binds to the barbed ends of actin filaments preventing further polymerization; localized predominantly to cortical actin patches
CAT2	YCAT	YML042	-	Carnitine acetyl-CoA transferase present in both mitochondria and peroxisomes, transfers activated acetyl groups to carnitine to form acetylcarnitine which can be shuttled across membranes
CAT8	DIL1|MSP8	YMR280	-	Zinc cluster transcriptional activator necessary for derepression of a variety of genes under non-fermentative growth conditions, active after diauxic shift, binds carbon source responsive elements
CCC2		YDR270	-	Cu(+2)-transporting P-type ATPase, required for export of copper from the cytosol into an extracytosolic compartment; has similarity to human proteins involved in Menkes and Wilsons diseases
CCH1		YGR217	+	Voltage-gated high-affinity calcium channel involved in calcium influx in response to some environmental stresses as well as exposure to mating pheromones; interacts and co-localizes with Mid1p, suggesting Cch1p and Mid1p function together
CCP1		YKR066	+	Mitochondrial cytochrome-c peroxidase; degrades reactive oxygen species in mitochondria, involved in the response to oxidative stress
CDC16		YKL022	+	Subunit of the anaphase-promoting complex/cyclosome (APC/C), which is a ubiquitin-protein ligase required for degradation of anaphase inhibitors, including mitotic cyclins, during the metaphase/anaphase transition; required for sporulation
CDC36	DNA19|NOT2	YDL165	+	Component of the CCR4-NOT complex, which has multiple roles in regulating mRNA levels including regulation of transcription and destabilizing mRNAs by deadenylation; basal transcription factor
CFT1	YHH1	YDR301	-	RNA-binding subunit of the mRNA cleavage and polyadenylation factor; involved in poly(A) site recognition and required for both pre-mRNA cleavage and polyadenylation, 51% sequence similarity with mammalian AAUAA-binding subunit of CPSF
CHA1		YCL064	-	Catabolic L-serine (L-threonine) deaminase, catalyzes the degradation of both L-serine and L-threonine; required to use serine or threonine as the sole nitrogen source, transcriptionally induced by serine and threonine
CHS6	CSD3	YJL099	+	Member of the ChAPs family of proteins (Chs5p-Arf1p-binding proteins: Bch1p, Bch2p, Bud7p, Chs6p), that forms the exomer complex with Chs5p to mediate export of specific cargo proteins, including Chs3p, from the Golgi to the plasma membrane
CIS1	ATG31	YDR022	-	Protein required for autophagosome formation in concert with Atg17p; may be involved in microtubule organization; high-copy suppressor of CIK1 deletion
CLB6		YGR109	-	B-type cyclin involved in DNA replication during S phase; activates Cdc28p to promote initiation of DNA synthesis; functions in formation of mitotic spindles along with Clb3p and Clb4p; most abundant during late G1
COS1		YNL336	-	Protein of unknown function, member of the DUP380 subfamily of conserved, often subtelomerically-encoded proteins

Gene Name	Alternate Gene Name	ORF	Up + or Down - regulation	Gene Description
COS10		YNR075	-	Protein of unknown function, member of the DUP380 subfamily of conserved, often subtelomerically-encoded proteins
COS12		YGL263	-	Protein of unknown function, member of the DUP380 subfamily of conserved, often subtelomerically-encoded proteins
COS6		YGR295	-	Protein of unknown function, member of the DUP380 subfamily of conserved, often subtelomerically-encoded proteins
COS7		YDL248	-	Protein of unknown function, member of the DUP380 subfamily of conserved, often subtelomerically-encoded proteins; the authentic, non-tagged protein is detected in highly purified mitochondria in high-throughput studies
COS8		YHL048	-	Nuclear membrane protein, member of the DUP380 subfamily of conserved, often subtelomerically-encoded proteins; regulation suggests a potential role in the unfolded protein response
COX1	OXI3	Q0045	+	Subunit I of cytochrome c oxidase, which is the terminal member of the mitochondrial inner membrane electron transport chain; one of three mitochondrially-encoded subunits
COX2	OXI1|OXII	Q0250	+	Subunit II of cytochrome c oxidase, which is the terminal member of the mitochondrial inner membrane electron transport chain; one of three mitochondrially-encoded subunits
COX7		YMR256	+	Subunit VII of cytochrome c oxidase, which is the terminal member of the mitochondrial inner membrane electron transport chain
COX8		YLR395	+	Subunit VIII of cytochrome c oxidase, which is the terminal member of the mitochondrial inner membrane electron transport chain
CRC1		YOR100	-	Mitochondrial inner membrane carnitine transporter, required for carnitine-dependent transport of acetyl-CoA from peroxisomes to mitochondria during fatty acid beta-oxidation
CRG1		YHR209	+	Putative S-adenosylmethionine-dependent methyltransferase; mediates cantharidin resistance
CSE4	CSL2	YKL049	+	Centromere protein that resembles histones, required for proper kinetochore function; homolog of human CENP-A
CUP1-2	CUP1	YHR055	+	Metallothionein, binds copper and mediates resistance to high concentrations of copper and cadmium; locus is variably amplified in different strains, with two copies, CUP1-1 and CUP1-2, in the genomic sequence reference strain S288C
CWP1	YJU1	YKL096	+	Cell wall mannoprotein, linked to a beta-1,3- and beta-1,6-glucan heteropolymer through a phosphodiester bond; involved in cell wall organization
CWP2	LPR1|YKL 097W-A	YKL096	+	Covalently linked cell wall mannoprotein, major constituent of the cell wall; plays a role in stabilizing the cell wall; involved in low pH resistance; precursor is GPI-anchored
DAD2	HSK1	YKR083	+	Essential subunit of the Dam1 complex (aka DASH complex), couples kinetochores to the force produced by MT depolymerization thereby aiding in chromosome segregation; is transferred to the kinetochore prior to mitosis
DAK2		YFL053	+	Dihydroxyacetone kinase, required for detoxification of dihydroxyacetone (DHA); involved in stress adaptation

Gene Name	Alternate Gene Name	ORF	Up + or Down - regulation	Gene Description
DAL2	ALC1	YIR029	-	Allantoicase, converts allantoate to urea and ureidoglycolate in the second step of allantoin degradation; expression sensitive to nitrogen catabolite repression and induced by allophanate, an intermediate in allantoin degradation
DAN3		YBR301	-	Cell wall mannoprotein with similarity to Tir1p, Tir2p, Tir3p, and Tir4p; expressed under anaerobic conditions, completely repressed during aerobic growth
DCG1		YIR030	-	Protein of unknown function, expression is sensitive to nitrogen catabolite repression and regulated by Dal80p; contains transmembrane domain
DCW1		YKL046	+	Putative mannosidase, GPI-anchored membrane protein required for cell wall biosynthesis in bud formation;homologous to Dfg5p
DDI2		YFL061	-	Protein whose expression is induced by DNA damage
DIA3		YDL024	-	Protein of unknown function, involved in invasive and pseudohyphal growth
DIC1		YLR348	-	Mitochondrial dicarboxylate carrier, integral membrane protein, catalyzes a dicarboxylate-phosphate exchange across the inner mitochondrial membrane, transports cytoplasmic dicarboxylates into the mitochondrial matrix
DID4	CHM2|GRD7|REN1|VPL2|VPS2|VPT14	YKL002	+	Class E Vps protein of the ESCRT-III complex, required for sorting of integral membrane proteins into lumenal vesicles of multivesicular bodies, and for delivery of newly synthesized vacuolar enzymes to the vacuole, involved in endocytosis
DIT1		YDR403	-	Sporulation-specific enzyme required for spore wall maturation, involved in the production of a soluble LL-dityrosine-containing precursor of the spore wall; transcripts accumulate at the time of prospore enclosure
DLD3		YEL071	-	D-lactate dehydrogenase, part of the retrograde regulon which consists of genes whose expression is stimulated by damage to mitochondria and reduced in cells grown with glutamate as the sole nitrogen source, located in the cytoplasm
DNF1		YER166	+	Aminophospholipid translocase (flippase) that localizes primarily to the plasma membrane; contributes to endocytosis, protein transport and cell polarity; type 4 P-type ATPase
DRE2		YKR071	+	Protein of unknown function; mutation displays synthetic lethal interaction with the pol3-13 allele of CDC2
EBP2		YKL172	-	Essential protein required for the maturation of 25S rRNA and 60S ribosomal subunit assembly, localizes to the nucleolus; constituent of 66S pre-ribosomal particles
ECI1		YLR284	-	Peroxisomal delta3,delta2-enoyl-CoA isomerase, hexameric protein that converts 3-hexenoyl-CoA to trans-2-hexenoyl-CoA, essential for the beta-oxidation of unsaturated fatty acids, oleate-induced
ECM18		YDR125	-	Protein of unknown function, similar to Rlp24p
ECM21		YBL101	-	Non-essential protein of unknown function; promoter contains several Gcn4p binding elements
ECM7	ZRG15	YLR443	+	Non-essential protein of unknown function
ELF1		YKL160	+	Transcription elongation factor that contains a conserved zinc finger domain; implicated in the maintenance of proper chromatin structure in actively transcribed regions; deletion inhibits Brome mosaic virus (BMV) gene expression

Gene Name	Alternate Gene Name	ORF	Up + or Down - regulation	Gene Description
ELP2	TOT2	YGR200	-	Subunit of Elongator complex, which is required for modification of wobble nucleosides in tRNA; target of Kluyveromyces lactis zymocin
EMP46		YLR080	-	Integral membrane component of endoplasmic reticulum-derived COPII-coated vesicles, which function in ER to Golgi transport
EMP47		YFL048	-	Integral membrane component of endoplasmic reticulum-derived COPII-coated vesicles, which function in ER to Golgi transport
ENA1	HOR6|PMR 2	YDR040	-	P-type ATPase sodium pump, involved in Na+ and Li+ efflux to allow salt tolerance
ENA2		YDR039	-	P-type ATPase sodium pump, involved in Na+ efflux to allow salt tolerance; likely not involved in Li+ efflux
ERD1	LDB2	YDR414	-	Predicted membrane protein required for the retention of lumenal endoplasmic reticulum proteins; mutants secrete the endogenous ER protein, BiP (Kar2p)
ERO1		YML130	+	Thiol oxidase required for oxidative protein folding in the endoplasmic reticulum
ERR1		YOR393	-	Protein of unknown function, has similarity to enolases
ERR2		YPL281	-	Protein of unknown function, has similarity to enolases
ERR3		YMR323	-	Protein of unknown function, has similarity to enolases
ERV15		YBR210	+	Protein involved in export of proteins from the endoplasmic reticulum, has similarity to Erv14p
ESF1		YDR365	-	Nucleolar protein involved in pre-rRNA processing; depletion causes severely decreased 18S rRNA levels
EST2	TERT	YLR318	-	Reverse transcriptase subunit of the telomerase holoenzyme, essential for telomerase core catalytic activity, involved in other aspects of telomerase assembly and function; mutations in human homolog are associated with aplastic anemia
EXG2		YDR261	-	Exo-1,3-beta-glucanase, involved in cell wall beta-glucan assembly; may be anchored to the plasma membrane via a glycosylphosphatidylinositol (GPI) anchor
EXG2		YDR261	-	Exo-1,3-beta-glucanase, involved in cell wall beta-glucan assembly; may be anchored to the plasma membrane via a glycosylphosphatidylinositol (GPI) anchor
FAR1		YJL157	-	Cyclin-dependent kinase inhibitor that mediates cell cycle arrest in response to pheromone; also forms a complex with Cdc24p, Ste4p, and Ste18p that may specify the direction of polarized growth during mating; potential Cdc28p substrate
FAR10		YLR238	-	Protein involved in G1 cell cycle arrest in response to pheromone, in a pathway different from the Far1p-dependent pathway; interacts with Far3p, Far7p, Far8p, Far9p, and Far11p; potential Cdc28p substrate
FAR3		YMR052	-	Protein involved in G1 cell cycle arrest in response to pheromone, in a pathway different from the Far1p-dependent pathway; interacts with Far7p, Far8p, Far9p, Far10p, and Far11p
FIG1		YBR040	-	Integral membrane protein required for efficient mating; may participate in or regulate the low affinity Ca2+ influx system, which affects intracellular signaling and cell-cell fusion during mating
FLO10		YKR102	+	Lectin-like protein with similarity to Flo1p, thought to be involved in flocculation
FLO8	PHD5|YER 108C	YER109	+	Transcription factor required for flocculation, diploid filamentous growth, and haploid invasive growth; genome reference strain S288C and most laboratory strains have a mutation in this gene

Gene Name	Alternate Gene Name	ORF	Up + or Down - regulation	Gene Description
FLR1		YBR008	+	Plasma membrane multidrug transporter of the major facilitator superfamily, involved in efflux of fluconazole, diazaborine, benomyl, methotrexate, and other drugs
FMP10		YER182	-	Putative protein of unknown function; the authentic, non-tagged protein is detected in highly purified mitochondria in high-throughput studies
FMP48		YGR052	+	Putative protein of unknown function; the authentic, non-tagged protein is detected in highly purified mitochondria in high-throughput studies
FMS1		YMR020	-	Polyamine oxidase, converts spermine to spermidine, which is required for the essential hypusination modification of translation factor eIF-5A; also involved in pantothenic acid biosynthesis
FMT1		YBL013	-	Methionyl-tRNA formyltransferase, catalyzes the formylation of initiator Met-tRNA in mitochondria; potential Cdc28p substrate
FRE2		YKL220	+	Ferric reductase and cupric reductase, reduces siderophore-bound iron and oxidized copper prior to uptake by transporters; expression induced by low iron levels but not by low copper levels
FRE4		YNR060	-	Ferric reductase, reduces a specific subset of siderophore-bound iron prior to uptake by transporters; expression induced by low iron levels
FSH1		YHR049	+	Serine hydrolase that localizes to both the nucleus and cytoplasm; sequence is similar to Fsh2p and Fsh3p
FUR4		YBR021	+	Uracil permease, localized to the plasma membrane; expression is tightly regulated by uracil levels and environmental cues
FUS1		YCL027	-	Membrane protein localized to the shmoo tip, required for cell fusion; expression regulated by mating pheromone; proposed to coordinate signaling, fusion, and polarization events required for fusion; potential Cdc28p substrate
FUS2		YMR232	+	Cytoplasmic protein localized to the shmoo tip; required for the alignment of parental nuclei before nuclear fusion during mating
FYV1		YDR024	+	Dubious open reading frame, unlikely to encode a protein; not conserved in closely related Saccharomyces species; mutation decreases survival upon exposure to K1 killer toxin
FYV12		YOR183	+	Protein of unknown function, required for survival upon exposure to K1 killer toxin
GAL1		YBR020	+	Galactokinase, phosphorylates alpha-D-galactose to alpha-D-galactose-1-phosphate in the first step of galactose catabolism; expression regulated by Gal4p
GAL10		YBR019	-	UDP-glucose-4-epimerase, catalyzes the interconversion of UDP-galactose and UDP-D-glucose in galactose metabolism; also catalyzes the conversion of alpha-D-glucose or alpha-D-galactose to their beta-anomers
GAL3		YDR009	-	Transcriptional regulator involved in activation of the GAL genes in response to galactose; forms a complex with Gal80p to relieve Gal80p inhibition of Gal4p; binds galactose and ATP but does not have galactokinase activity
GAL4	GAL81	YPL248	-	DNA-binding transcription factor required for the activation of the GAL genes in response to galactose; repressed by Gal80p and activated by Gal3p
GAL7		YBR018	-	Galactose-1-phosphate uridyl transferase, synthesizes glucose-1-phosphate and UDP-galactose from UDP-D-glucose and alpha-D-galactose-1-phosphate in the second step of galactose catabolism
GAS5		YOL030	+	1,3-beta-glucanosyltransferase, has similarity to Gas1p; localizes to the cell wall

Gene Name	Alternate Gene Name	ORF	Up + or Down - regulation	Gene Description
GCN4	AAS3|ARG 9	YEL009	+	Transcriptional activator of amino acid biosynthetic genes in response to amino acid starvation; expression is tightly regulated at both the transcriptional and translational levels
GCR2		YNL199	-	Transcriptional activator of genes involved in glycolysis; interacts and functions with the DNA-binding protein Gcr1p
GCV3		YAL044	+	H subunit of the mitochondrial glycine decarboxylase complex, required for the catabolism of glycine to 5,10-methylene-THF; expression is regulated by levels of levels of 5,10-methylene-THF in the cytoplasm
GDH3	FUN51	YAL062	-	NADP(+)-dependent glutamate dehydrogenase, synthesizes glutamate from ammonia and alpha-ketoglutarate; rate of alpha-ketoglutarate utilization differs from Gdh1p; expression regulated by nitrogen and carbon sources
GFA1		YKL104	+	Glutamine-fructose-6-phosphate amidotransferase, catalyzes the formation of glucosamine-6-P and glutamate from fructose-6-P and glutamine in the first step of chitin biosynthesis
GIT1		YCR098	-	Plasma membrane permease, mediates uptake of glycerophosphoinositol and glycerophosphocholine as sources of the nutrients inositol and phosphate; expression and transport rate are regulated by phosphate and inositol availability
GLE2	RAE1	YER107	-	Component of the nuclear pore complex required for polyadenylated RNA export but not for protein import, homologous to S. pombe Rae1p
GLK1	HOR3	YCL040	-	Glucokinase, catalyzes the phosphorylation of glucose at C6 in the first irreversible step of glucose metabolism; one of three glucose phosphorylating enzymes; expression regulated by non-fermentable carbon sources
GLO4		YOR040	-	Mitochondrial glyoxalase II, catalyzes the hydrolysis of S-D-lactoylglutathione into glutathione and D-lactate
GND2		YGR256	-	6-phosphogluconate dehydrogenase (decarboxylating), catalyzes an NADPH regenerating reaction in the pentose phosphate pathway; required for growth on D-glucono-delta-lactone
GPD2	GPD3	YOL059	-	NAD-dependent glycerol 3-phosphate dehydrogenase, homolog of Gpd1p, expression is controlled by an oxygen-independent signaling pathway required to regulate metabolism under anoxic conditions; located in cytosol and mitochondria
GPI10		YGL142	+	Integral membrane protein involved in glycosylphosphatidylinositol (GPI) anchor synthesis; putative alpha 1,2 mannosyltransferase required for addition of the third mannose onto the GPI core structure; human PIG-Bp is a functional homolog
GPI11		YDR302	-	ER membrane protein involved in a late step of glycosylphosphatidylinositol (GPI) anchor assembly; involved in the addition of phosphoethanolamine to the multiply mannosylated GPI intermediate; human PIG-Fp is a functional homolog
GPM2		YDL021	-	Homolog of Gpm1p phosphoglycerate mutase which converts 3-phosphoglycerate to 2-phosphoglycerate in glycolysis; may be non-functional derivative of a gene duplication event
GRC3		YLL035	+	Protein of unknown function, required for cell growth and possibly involved in rRNA processing; mRNA is cell cycle regulated
GRX3		YDR098	-	Hydroperoxide and superoxide-radical responsive glutathione-dependent oxidoreductase; monothiol glutaredoxin subfamily member along with Grx4p and Grx5p; protects cells from oxidative damage

Gene Name	Alternate Gene Name	ORF	Up + or Down - regulation	Gene Description
HAP1	CYP1	YLR256	-	Zinc finger transcription factor involved in the complex regulation of gene expression in response to levels of heme and oxygen; the S288C sequence differs from other strain backgrounds due to a Ty1 insertion in the carboxy terminus
HCS1	DIP1	YKL017	+	Hexameric DNA polymerase alpha-associated DNA helicase A involved in lagging strand DNA synthesis; contains single-stranded DNA stimulated ATPase and dATPase activities; replication protein A stimulates helicase and ATPase activities
HDA1		YNL021	-	Putative catalytic subunit of a class II histone deacetylase complex that also contains Hda2p and Hda3p; Hda1p interacts with the Hda2p-Hda3p subcomplex to form an active tetramer; deletion increases histone H2B, H3 and H4 acetylation
HEF3	ZRG7	YNL014	-	Translational elongation factor EF-3; paralog of YEF3 and member of the ABC superfamily; stimulates EF-1 alpha-dependent binding of aminoacyl-tRNA by the ribosome; normally expressed in zinc deficient cells
HEM12	HEM6	YDR047	-	Uroporphyrinogen decarboxylase, catalyzes the fifth step in the heme biosynthetic pathway; localizes to both the cytoplasm and nucleus; activity inhibited by Cu2+, Zn2+, Fe2+, Fe3+ and sulfhydryl-specific reagents
HEM2	SLU1	YGL040	-	Delta-aminolevulinate dehydratase, a homo-octameric enzyme, catalyzes the conversion of delta-aminolevulinic acid to porphobilinogen, the second step in the heme biosynthetic pathway; localizes to both the cytoplasm and nucleus
HEM4	SLU2	YOR278	+	Uroporphyrinogen III synthase, catalyzes the conversion of hydroxymethylbilane to uroporphyrinogen III, the fourth step in the heme biosynthetic pathway
HGH1		YGR187	-	Protein of unknown function with similarity to human HMG1 and HMG2; localizes to the cytoplasm
HIT1		YJR055	-	Protein of unknown function, required for growth at high temperature
HOS1		YPR068	-	Putative class I histone deacetylase (HDAC) with sequence similarity to Hda1p, Rpd3p, Hos2p, and Hos3p; deletion results in increased histone acetylation at rDNA repeats; interacts with the Tup1p-Ssn6p corepressor complex
HPF1		YOL155	+	Haze-protective mannoprotein that reduces the particle size of aggregated proteins in white wines
HSL1	NIK1	YKL101	+	Nim1p-related protein kinase that regulates the morphogenesis and septin checkpoints; associates with the assembled septin filament; required along with Hsl7p for bud neck recruitment, phosphorylation, and degradation of Swe1p
HSP150	CCW7|ORE1|PIR2	YJL159	+	O-mannosylated heat shock protein that is secreted and covalently attached to the cell wall via beta-1,3-glucan and disulfide bridges; required for cell wall stability; induced by heat shock, oxidative stress, and nitrogen limitation
HSP31		YDR533	+	Possible chaperone and cysteine protease with similarity to E. coli Hsp31; member of the DJ-1/ThiJ/PfpI superfamily, which includes human DJ-1 involved in Parkinson's disease; exists as a dimer and contains a putative metal-binding site
HSP32		YPL280	-	Possible chaperone and cysteine protease with similarity to E. coli Hsp31 and S. cerevisiae Hsp31p, Hsp33p, and Sno4p; member of the DJ-1/ThiJ/PfpI superfamily, which includes human DJ-1 involved in Parkinson's disease

Gene Name	Alternate Gene Name	ORF	Up + or Down - regulation	Gene Description
HSP33		YOR391	-	Enzyme of 'de novo' purine biosynthesis containing both 5-aminoimidazole-4-carboxamide ribonucleotide transformylase and inosine monophosphate cyclohydrolase activities, isozyme of Ade16p; ade16 ade17 mutants require adenine and histidine
HST4		YDR191	-	Member of the Sir2 family of NAD(+)-dependent protein deacetylases; involved along with Hst3p in silencing at telomeres, cell cycle progression, radiation resistance, genomic stability and short-chain fatty acid metabolism
HTS1	TSM4572	YPR033	-	Cytoplasmic and mitochondrial histidine tRNA synthetase; encoded by a single nuclear gene that specifies two messages; efficient mitochondrial localization requires both a presequence and an amino-terminal sequence
HUA2		YOR284	-	Cytoplasmic protein of unknown function; computational analysis of large-scale protein-protein interaction data suggests a possible role in actin patch assembly
HVG1	YEM9	YER039	-	Protein of unknown function, has homology to Vrg4p
HXT13		YEL069	+	Hexose transporter, induced in the presence of non-fermentable carbon sources, induced by low levels of glucose, repressed by high levels of glucose
HXT15		YDL245	+	Protein of unknown function with similarity to hexose transporter family members, expression is induced by low levels of glucose and repressed by high levels of glucose
HXT17		YNR072	+	Hexose transporter, up-regulated in media containing raffinose and galactose at pH 7.7 versus pH 4.7, repressed by high levels of glucose
ICY1		YMR195	-	Protein of unknown function, required for viability in rich media of cells lacking mitochondrial DNA; mutants have an invasive growth defect with elongated morphology; induced by amino acid starvation
IDH1		YNL037	-	Subunit of mitochondrial NAD(+)-dependent isocitrate dehydrogenase, which catalyzes the oxidation of isocitrate to alpha-ketoglutarate in the TCA cycle
IML1		YJR138	+	Protein of unknown function, green fluorescent protein (GFP)-fusion protein localizes to the vacuolar membrane
IMP2'	IMP2	YIL154	-	Transcriptional activator involved in maintenance of ion homeostasis and protection against DNA damage caused by bleomycin and other oxidants, contains a C-terminal leucine-rich repeat
IRC11		YOR013	-	Hypothetical protein; null mutant displays increased levels of spontaneous Rad52 foci
IRC13		YOR235	-	Hypothetical protein; null mutant displays increased levels of spontaneous Rad52 foci
IRC4		YDR540	-	Hypothetical protein; null mutant displays increased levels of spontaneous Rad52 foci
ITR2	HRB612	YOL103	-	Myo-inositol transporter with strong similarity to the major myo-inositol transporter Itr1p, member of the sugar transporter superfamily; expressed constitutively
ITT1		YML068	+	Protein that modulates the efficiency of translation termination, interacts with translation release factors eRF1 (Sup45p) and eRF3 (Sup35p) in vitro, contains a zinc finger domain characteristic of the TRIAD class of proteins
IZH4		YOL101	+	Membrane protein involved in zinc metabolism, member of the four-protein IZH family, expression induced by fatty acids and altered zinc levels; deletion reduces sensitivity to excess zinc; possible role in sterol metabolism

Gene Name	Alternate Gene Name	ORF	Up + or Down - regulation	Gene Description
JIP4	YDR474C	YDR475	-	Protein of unknown function; previously annotated as two separate ORFs, YDR474C and YDR475C, which were merged as a result of corrections to the systematic reference sequence
JLP1		YLL057	-	Fe(II)-dependent sulfonate/alpha-ketoglutarate dioxygenase, involved in sulfonate catabolism for use as a sulfur source, contains sequence that closely resembles a J domain (typified by the E. coli DnaJ protein)
JNM1	INS1|PAC3	YMR294	-	Component of the yeast dynactin complex, consisting of Nip100p, Jnm1p, and Arp1p; required for proper nuclear migration and spindle partitioning during mitotic anaphase B
KAE1		YKR038	+	Putative glycoprotease proposed to be in transcription as a component of the EKC protein complex with Bud32p, Cgi121p, Pcc1p, and Gon7p; also identified as a component of the KEOPS protein complex
KAP114		YGL241	-	Karyopherin, responsible for nuclear import of Spt15p, histones H2A and H2B, and Nap1p; amino terminus shows similarity to those of other importins, particularly Cse1p; localization is primarily nuclear
KAR5	FIG3	YMR065	-	Protein required for nuclear membrane fusion during karyogamy, localizes to the membrane with a soluble portion in the endoplasmic reticulum lumen, may form a complex with Jem1p and Kar2p; expression of the gene is regulated by pheromone
KRE11	TRS65	YGR166	+	Protein involved in biosynthesis of cell wall beta-glucans; subunit of the TRAPP (transport protein particle) complex, which is involved in the late steps of endoplasmic reticulum to Golgi transport
KTI11	DPH3	YBL071	+	Zn-ribbon electron carrier protein, required with Dph1p, Dph2p, Jjj3p, and Dph5p for synthesis of diphthamide, a modified histidine residue on Eft1p or Eft2p; required, with Elongator complex, for modification of wobble nucleosides in tRNA
KTI12	TOT4	YKL110	+	Protein that plays a role, with Elongator complex, in modification of wobble nucleosides in tRNA; involved in sensitivity to G1 arrest induced by zymocin; interacts with chromatin throughout the genome; also interacts with Cdc19p
KTR2		YKR061	+	Mannosyltransferase involved in N-linked protein glycosylation; member of the KRE2/MNT1 mannosyltransferase family
LAG2	ECM36	YOL025	-	Protein involved in determination of longevity; LAG2 gene is preferentially expressed in young cells; overexpression extends the mean and maximum life span of cells
LAS1		YKR063	+	Essential nuclear protein possibly involved in bud formation and morphogenesis; mutants require the SSD1-v allele for viability
LEE1		YPL054	-	Zinc-finger protein of unknown function
LEU2		YCL007	+	Beta-isopropylmalate dehydrogenase (IMDH), catalyzes the third step in the leucine biosynthesis pathway
LEU9		YOR108	+	Alpha-isopropylmalate synthase II (2-isopropylmalate synthase), catalyzes the first step in the leucine biosynthesis pathway; the minor isozyme, responsible for the residual alpha-IPMS activity detected in a leu4 null mutant
LHS1	CER1|SSI1	YKL073	+	Molecular chaperone of the endoplasmic reticulum lumen, involved in polypeptide translocation and folding; member of the Hsp70 family; localizes to the lumen of the ER; regulated by the unfolded protein response pathway

Gene Name	Alternate Gene Name	ORF	Up + or Down - regulation	Gene Description
LIA1	MMD1	YJR070	-	Deoxyhypusine hydroxylase, a HEAT-repeat containing metalloenzyme that catalyses hypusine formation; binds to and is required for the modification of Hyp2p (eIF5A); complements S. pombe mmd1 mutants defective in mitochondrial positioning
LOT5		YKL183	+	Protein of unknown function; gene expression increases in cultures shifted to a lower temperature
LPE10		YPL060	-	Mitochondrial inner membrane magnesium transporter, involved in maintenance of magnesium concentrations inside mitochondria; indirectly affects splicing of group II introns; functionally and structurally related to Mrs2p
LRC3		YKL207	+	Putative protein of unknown function; non-essential gene; null mutant displays decreased frequency of mitochondrial genome loss (petite formation) and severe growth defect in minimal glycerol media
LRC5	FMP38	YOR205	-	Protein of unknown function; non-tagged protein is detected in purified mitochondria in high-throughput studies; null mutant displays decreased frequency of mitochondrial genome loss and severe growth defect in minimal glycerol media
LRS4		YDR439	-	Protein involved in rDNA silencing; positively charged coiled-coil protein with limited similarity to myosin
LSC1		YOR142	-	Alpha subunit of succinyl-CoA ligase, which is a mitochondrial enzyme of the TCA cycle that catalyzes the nucleotide-dependent conversion of succinyl-CoA to succinate; phosphorylated
LST4		YKL176	+	Protein possibly involved in a post-Golgi secretory pathway; required for the transport of nitrogen-regulated amino acid permease Gap1p from the Golgi to the cell surface
LUG1		YCR087	+	Putative protein of unknown function; green fluorescent protein (GFP)-fusion protein localizes to the nucleolus; YCR087C-A is not an essential gene
LYS1		YIR034	+	Saccharopine dehydrogenase (NAD+, L-lysine-forming), catalyzes the conversion of saccharopine to L-lysine, which is the final step in the lysine biosynthesis pathway
LYS14		YDR034	-	Transcriptional activator involved in regulation of genes of the lysine biosynthesis pathway; requires 2-aminoadipate semialdehyde as co-inducer
MAG1	MMS5	YER142	-	3-methyl-adenine DNA glycosylase involved in protecting DNA against alkylating agents; initiates base excision repair by removing damaged bases to create abasic sites that are subsequently repaired
MAK11		YKL021	+	Protein involved in an early, nucleolar step of 60S ribosomal subunit biogenesis; essential for cell growth and replication of killer M1 dsRNA virus; contains four beta-transducin repeats
MAL12	MALS	YGR292	-	Maltase (alpha-D-glucosidase), inducible protein involved in maltose catabolism; encoded in the MAL1 complex locus
MAL32	MAL3S|MALS	YBR299	-	Maltase (alpha-D-glucosidase), inducible protein involved in maltose catabolism; encoded in the MAL3 complex locus; functional in genomic reference strain S288C
MCH5		YOR306	+	Plasma membrane riboflavin transporter; facilitates the uptake of vitamin B2; required for FAD-dependent processes; sequence similarity to mammalian monocarboxylate permeases, however mutants are not deficient in monocarboxylate transport
MCM2		YBL023	-	Protein involved in DNA replication; component of the Mcm2-7 hexameric complex that binds chromatin as a part of the pre-replicative complex

Gene Name	Alternate Gene Name	ORF	Up + or Down - regulation	Gene Description
MDH1		YKL085	+	Mitochondrial malate dehydrogenase, catalyzes interconversion of malate and oxaloacetate; involved in the tricarboxylic acid (TCA) cycle; phosphorylated
MDJ2		YNL328	-	Constituent of the mitochondrial import motor associated with the presequence translocase; function overlaps with that of Pam18p; stimulates the ATPase activity of Ssc1p to drive mitochondrial import; contains a J domain
MDM35		YKL053	+	Mitochondrial intermembrane space cysteine motif protein; mutation affects mitochondrial distribution and morphology
MEC1	ESR1|SAD3	YBR113	+	Genome integrity checkpoint protein and PI kinase superfamily member; signal transducer required for cell cycle arrest and transcriptional responses prompted by damaged or unreplicated DNA; monitors and participates in meiotic recombination
MEF2		YJL102	+	Mitochondrial elongation factor involved in translational elongation
MEI5	LPH6	YPL121	+	Meiosis specific protein involved in DMC1-dependent meiotic recombination, forms heterodimer with Sae3p; proposed to be an assembly factor for Dmc1p
MET12		YPL023	+	Isozyme of methylenetetrahydrofolate reductase, catalyzes the reduction of 5,10-methylenetetrahydrofolate to 5-methyltetrahydrofolate in the methionine biosynthesis pathway
MET17	MET15|MET25	YLR303	-	Methionine and cysteine synthase (O-acetyl homoserine-O-acetyl serine sulfhydrylase), required for sulfur amino acid synthesis
MET22	HAL2	YOL064	-	Bisphosphate-3'-nucleotidase, involved in salt tolerance and methionine biogenesis; dephosphorylates 3'-phosphoadenosine-5'-phosphate and 3'-phosphoadenosine-5'-phosphosulfate, intermediates of the sulfate assimilation pathway
MET6		YER091	-	Cobalamin-independent methionine synthase, involved in amino acid biosynthesis; requires a minimum of two glutamates on the methyltetrahydrofolate substrate, similar to bacterial metE homologs
MGM101	MGM9	YJR144	+	Protein involved in mitochondrial genome maintenance; component of the mitochondrial nucleoid, required for the repair of oxidative mtDNA damage
MHT1		YLL062	-	S-methylmethionine-homocysteine methyltransferase, functions along with Sam4p in the conversion of S-adenosylmethionine (AdoMet) to methionine to control the methionine/AdoMet ratio
MIC14		YDR031	-	Mitochondrial intermembrane space cysteine motif protein of 14 kDa
MIF2		YKL089	+	Kinetochore protein with homology to human CENP-C, required for structural integrity of the spindle during anaphase spindle elongation, interacts with histones H2A, H2B, and H4, phosphorylated by Ipl1p
MIM1	TOM13	YOL026	+	Mitochondrial outer membrane protein, required for assembly of the translocase of the outer membrane (TOM) complex and thereby for mitochondrial protein import; N terminus is exposed to the cytosol: transmembrane segment is highly conserved
MIP6		YHR015	-	Putative RNA-binding protein, interacts with Mex67p, which is a component of the nuclear pore involved in nuclear mRNA export
MLH2		YLR035	-	Protein involved in the mismatch repair of certain frameshift intermediates and involved in meiotic recombination; forms a complex with Mlh1p
MMP1		YLL061	-	High-affinity S-methylmethionine permease, required for utilization of S-methylmethionine as a sulfur source; has similarity to S-adenosylmethionine permease Sam3p

Gene Name	Alternate Gene Name	ORF	Up + or Down - regulation	Gene Description
MMS22	SLM2	YLR320	+	Protein involved in resistance to ionizing radiation; acts with Mms1p in a repair pathway that may be involved in resolving replication intermediates or preventing the damage caused by blocked replication forks
MNR2		YKL064	+	Putative magnesium transporter; has similarity to Alr1p and Alr2p, which mediate influx of Mg2+ and other divalent cations
MNT3		YIL014	-	Alpha-1,3-mannosyltransferase, adds the fourth and fifth alpha-1,3-linked mannose residues to O-linked glycans during protein O-glycosylation
MOB1		YIL106	-	Component of the mitotic exit network; associates with and is required for the activation and Cdc15p-dependent phosphorylation of the Dbf2p kinase; required for cytokinesis and cell separation; component of the CCR4 transcriptional complex
MPC54		YOR177	-	Component of the meiotic outer plaque, a membrane-organizing center which is assembled on the cytoplasmic face of the spindle pole body during meiosis II and triggers the formation of the prospore membrane; potential Cdc28p substrate
MRE11	NGS1|RAD58|XRS4	YMR224	-	Subunit of a complex with Rad50p and Xrs2p (RMX complex) that functions in repair of DNA double-strand breaks and in telomere stability, exhibits nuclease activity that appears to be required for RMX function; widely conserved
MRH1		YDR033	-	Protein that localizes primarily to the plasma membrane, also found at the nuclear envelope; the authentic, non-tagged protein is detected in mitochondria in a phosphorylated state; has similarity to Hsp30p and Yro2p
MRN1		YPL184	+	Putative RNA binding protein
MRPL10	MRPL18	YNL284	-	Mitochondrial ribosomal protein of the large subunit; appears as two protein spots (YmL10 and YmL18) on two-dimensional SDS gels
MRPL44	YMR44	YMR225	+	Mitochondrial ribosomal protein of the large subunit
MSH3		YCR092	+	Mismatch repair protein, forms dimers with Msh2p that mediate repair of insertion or deletion mutations and removal of nonhomologous DNA ends, contains a PCNA (Pol30p) binding motif required for genome stability
MSH4		YFL003	-	Protein involved in meiotic recombination, required for normal levels of crossing over, colocalizes with Zip2p to discrete foci on meiotic chromosomes, has homology to bacterial MutS protein
MSP1	YTA4	YGR028	-	Mitochondrial protein involved in sorting of proteins in the mitochondria; putative membrane-spanning ATPase
MSS1	PET53	YMR023	-	Mitochondrial protein, forms a heterodimer complex with Mto1p that performs the 5-carboxymethylaminomethyl modification of the wobble uridine base in mitochondrial tRNAs; similar to human GTPBP3
MST1		YKL194	+	Mitochondrial threonyl-tRNA synthetase
MST28		YAR033	-	Putative integral membrane protein, involved in vesicle formation; forms complex with Mst27p; member of DUP240 gene family; binds COPI and COPII vesicles

Gene Name	Alternate Gene Name	ORF	Up + or Down - regulation	Gene Description
MTD1		YKR080	+	NAD-dependent 5,10-methylenetetrahydrafolate dehydrogenase, plays a catalytic role in oxidation of cytoplasmic one-carbon units; expression is regulated by Bas1p and Bas2p, repressed by adenine, and may be induced by inositol and choline
MTF1		YMR228	-	Mitochondrial RNA polymerase specificity factor with structural similarity to S-adenosylmethionine-dependent methyltransferases and functional similarity to bacterial sigma-factors, interacts with mitochondrial core polymerase Rpo41p
MUC1	FLO11|STA4	YIR019	+	GPI-anchored cell surface glycoprotein (flocculin) required for pseudohyphal formation, invasive growth, flocculation, and biofilms; transcriptionally regulated by the MAPK pathway (via Ste12p and Tec1p) and the cAMP pathway (via Flo8p)
NDE2	NDH2	YDL085	-	Mitochondrial external NADH dehydrogenase, catalyzes the oxidation of cytosolic NADH; Nde1p and Nde2p are involved in providing the cytosolic NADH to the mitochondrial respiratory chain
NDJ1	TAM1	YOL104	-	Meiosis-specific telomere protein, required for bouquet formation, effective homolog pairing, ordered cross-over distribution (interference), sister chromatid cohesion at meiotic telomeres, and segregation of small chromosomes
NFT1		YKR103	+	Putative transporter of the multidrug resistance-associated protein (MRP) subfamily; adjacent ORFs YKR103W and YKR104W are merged in different strain backgrounds.
NFU1	NUB1	YKL040	+	Protein involved in iron metabolism in mitochondria; similar to NifU, which is a protein required for the maturation of the Fe/S clusters of nitrogenase in nitrogen-fixing bacteria
NGL3		YML118	-	Putative endonuclease, has a domain similar to a magnesium-dependent endonuclease motif in mRNA deadenylase Ccr4p; similar to Ngl1p and Ngl2p
NKP1		YDR383	-	Non-essential kinetochore protein, subunit of the Ctf19 central kinetochore complex (Ctf19p-Mcm21p-Okp1p-Mcm22p-Mcm16p-Ctf3p-Chl4p-Mcm19p-Nkp1p-Nkp2p-Ame1p-Mtw1p)
NOP4	NOP77	YPL043	-	Nucleolar protein, essential for processing and maturation of 27S pre-rRNA and large ribosomal subunit biogenesis; constituent of 66S pre-ribosomal particles; contains four RNA recognition motifs (RRMs)
NQM1		YGR043	-	Protein of unknown function; transcription is repressed by Mot1p and induced by alpha-factor and during diauxic shift; null mutant non-quiescent cells exhibit reduced reproductive capacity
NRD1		YNL251	+	RNA-binding protein that interacts with the C-terminal domain of the RNA polymerase II large subunit (Rpo21p), required for transcription termination and 3' end maturation of nonpolyadenylated RNAs
NSA1		YGL111	-	Constituent of 66S pre-ribosomal particles, involved in 60S ribosomal subunit biogenesis
NSE3		YDR285	-	Essential subunit of the Mms21-Smc5-Smc6 complex; protein of unknown function; required for DNA repair and growth
NSL1		YPL233	-	Essential component of the MIND kinetochore complex (Mtw1p Including Nnf1p-Nsl1p-Dsn1p) which joins kinetochore subunits contacting DNA to those contacting microtubules; required for accurate chromosome segregation
NTE1		YML059	-	Serine esterase that deacylates exogenous lysophospholipids, homolog of human neuropathy target esterase (NTE); mammalian NTE1 deacylates phosphatidylcholine to glycerophosphocholine
NTH1		YDR001	-	Neutral trehalase, degrades trehalose; required for thermotolerance and may mediate resistance to other cellular stresses; may be phosphorylated by Cdc28p

Gene Name	Alternate Gene Name	ORF	Up + or Down - regulation	Gene Description
NTR2		YKR022	+	Essential protein that forms a dimer with Ntr1p; also forms a trimer, with Ntr2p and the DExD/H-box RNA helicase Prp43p, that is involved in spliceosome disassembly
NUG1		YER006	-	GTPase that associates with nuclear 60S pre-ribosomes, required for export of 60S ribosomal subunits from the nucleus
NUP133	RAT3	YKR082	-	Subunit of the Nup84p subcomplex of the nuclear pore complex (NPC), localizes to both sides of the NPC, required to establish a normal nucleocytoplasmic concentration gradient of the GTPase Gsp1p
NUP188		YML103	+	Subunit of the nuclear pore complex (NPC), involved in the structural organization of the complex and of the nuclear envelope, also involved in nuclear envelope permeability, interacts with Pom152p and Nic96p
OAF3		YKR064	+	Zinc cluster protein of unknown function; the authentic, non-tagged protein is detected in highly purified mitochondria in high-throughput studies
OM45		YIL136	-	Protein of unknown function, major constituent of the mitochondrial outer membrane; located on the outer (cytosolic) face of the outer membrane
OMA1		YKR087	+	Metalloendopeptidase of the mitochondrial inner membrane, involved in turnover of membrane-embedded proteins; member of a family of predicted membrane-bound metallopeptidases in prokaryotes and higher eukaryotes
OMS1		YDR316	-	Protein integral to the mitochondrial membrane; has a conserved methyltransferase motif; multicopy suppressor of respiratory defects caused by OXA1 mutations
OMS1		YDR316	-	Protein integral to the mitochondrial membrane; has a conserved methyltransferase motif; multicopy suppressor of respiratory defects caused by OXA1 mutations
ORM1		YGR038	-	Evolutionarily conserved protein with similarity to Orm2p, required for resistance to agents that induce the unfolded protein response; human ortholog is located in the endoplasmic reticulum
OST5		YGL226	+	Zeta subunit of the oligosaccharyltransferase complex of the ER lumen, which catalyzes asparagine-linked glycosylation of newly synthesized proteins
OSW2		YLR054	-	Protein of unknown function proposed to be involved in the assembly of the spore wall
OYE3	ZRG6	YPL171	+	Widely conserved NADPH oxidoreductase containing flavin mononucleotide (FMN), homologous to Oye2p with slight differences in ligand binding and catalytic properties; may be involved in sterol metabolism
PAM1		YDR251	-	Essential protein of unknown function; exhibits variable expression during colony morphogenesis; overexpression permits survival without protein phosphatase 2A, inhibits growth, and induces a filamentous phenotype
PAM17	FMP18	YKR065	+	Presequence translocase-associated motor subunit, required for stable complex formation between cochaperones Pam16p and Pam18p, promotes association of Pam16p-Pam18p with the presequence translocase
PAN1	DIM2|MDP3|MIP3	YIR006	-	Part of actin cytoskeleton-regulatory complex Pan1p-Sla1p-End3p, associates with actin patches on the cell cortex; promotes protein-protein interactions essential for endocytosis; previously thought to be a subunit of poly(A) ribonuclease
PAN3	ECM35	YKL025	+	Essential subunit of the Pan2p-Pan3p poly(A)-ribonuclease complex, which acts to control poly(A) tail length and regulate the stoichiometry and activity of postreplication repair complexes

Gene Name	Alternate Gene Name	ORF	Up + or Down - regulation	Gene Description
PAU1		YJL223	-	Part of 23-member seripauperin multigene family encoded mainly in subtelomeric regions, active during alcoholic fermentation, regulated by anaerobiosis, negatively regulated by oxygen, repressed by heme
PAU10		YDR542	-	Hypothetical protein
PAU11		YGL261	-	Putative protein of unknown function; mRNA expression appears to be regulated by SUT1 and UPC2
PAU12		YGR294	-	Hypothetical protein
PAU13		YHL046	-	Putative protein of unknown function; not an essential gene
PAU14		YIL176	-	Hypothetical protein
PAU16		YKL224	-	Putative protein of unknown function
PAU18		YLL064	-	Hypothetical protein
PAU19		YMR325	-	Hypothetical protein
PAU20		YOL161	-	Hypothetical protein
PAU21		YOR394	-	Hypothetical protein
PAU22		YPL282	-	Hypothetical protein
PAU3		YCR104	-	Part of 23-member seripauperin multigene family encoded mainly in subtelomeric regions, active during alcoholic fermentation, regulated by anaerobiosis, negatively regulated by oxygen, repressed by heme
PAU4		YLR461	-	Part of 23-member seripauperin multigene family encoded mainly in subtelomeric regions, active during alcoholic fermentation, regulated by anaerobiosis, negatively regulated by oxygen, repressed by heme
PAU6		YNR076	-	Part of 23-member seripauperin multigene family encoded mainly in subtelomeric regions, active during alcoholic fermentation, regulated by anaerobiosis, negatively regulated by oxygen, repressed by heme
PAU8		YAL068	-	Hypothetical protein
PBS2	HOG4|SFS4|SSK4	YJL128	-	MAP kinase kinase that plays a pivotal role in the osmosensing signal-transduction pathway, activated under severe osmotic stress
PDE1		YGL248	-	Low-affinity cyclic AMP phosphodiesterase, controls glucose and intracellular acidification-induced cAMP signaling, target of the cAMP-protein kinase A (PKA) pathway; glucose induces transcription and inhibits translation
PDH1		YPR002	-	Mitochondrial protein that participates in respiration, induced by diauxic shift; homologous to E. coli PrpD, may take part in the conversion of 2-methylcitrate to 2-methylisocitrate
PDH1		YPR002	-	Mitochondrial protein that participates in respiration, induced by diauxic shift; homologous to E. coli PrpD, may take part in the conversion of 2-methylcitrate to 2-methylisocitrate
PDR15		YDR406	-	Plasma membrane ATP binding cassette (ABC) transporter, multidrug transporter and general stress response factor implicated in cellular detoxification; regulated by Pdr1p, Pdr3p and Pdr8p; promoter contains a PDR responsive element
PDR3	AMY2|TPE2	YBL005	-	Transcriptional activator of the pleiotropic drug resistance network, regulates expression of ATP-binding cassette (ABC) transporters through binding to cis-acting sites known as PDREs (PDR responsive elements)
PDR5	LEM1|STS1|YDR1	YOR153	-	Plasma membrane ATP-binding cassette (ABC) transporter, short-lived multidrug transporter actively regulated by Pdr1p; also involved in steroid transport, cation resistance, and cellular detoxification during exponential growth

Gene Name	Alternate Gene Name	ORF	Up + or Down - regulation	Gene Description
PEP5	END1|VAM1|VPL9|VPS11|VPT11	YMR231	+	Component of CORVET tethering complex; peripheral vacuolar membrane protein required for protein trafficking and vacuole biogenesis; interacts with Pep7p
PET10		YKR046	+	Protein of unknown function that co-purifies with lipid particles; expression pattern suggests a role in respiratory growth; computational analysis of large-scale protein-protein interaction data suggests a role in ATP/ADP exchange
PFK1		YGR240	-	Alpha subunit of heterooctameric phosphofructokinase involved in glycolysis, indispensable for anaerobic growth, activated by fructose-2,6-bisphosphate and AMP, mutation inhibits glucose induction of cell cycle-related genes
PGA2		YNL149	+	Essential protein required for maturation of Gas1p and Pho8p; involved in protein trafficking; GFP-fusion protein localizes to the ER and YFP-fusion protein to the nuclear envelope-ER network; null mutants have a cell separation defect
PHO11		YAR071	-	One of three repressible acid phosphatases, a glycoprotein that is transported to the cell surface by the secretory pathway; induced by phosphate starvation and coordinately regulated by PHO4 and PHO2
PHO12	PHO10	YHR215	-	Glycerol proton symporter of the plasma membrane, subject to glucose-induced inactivation, strongly but transiently induced when cells are subjected to osmotic shock
PHO84		YML123	-	High-affinity inorganic phosphate (Pi) transporter and low-affinity manganese transporter; regulated by Pho4p and Spt7p; mutation confers resistance to arsenate; exit from the ER during maturation requires Pho86p
PHO89	ITN1	YBR296	-	Na+/Pi cotransporter, active in early growth phase; similar to phosphate transporters of Neurospora crassa; transcription regulated by inorganic phosphate concentrations and Pho4p
PIR1	CCW6	YKL164	+	O-glycosylated protein required for cell wall stability; attached to the cell wall via beta-1,3-glucan; mediates mitochondrial translocation of Apn1p; expression regulated by the cell integrity pathway and by Swi5p during the cell cycle
PIR3	CCW8	YKL163	+	O-glycosylated covalently-bound cell wall protein required for cell wall stability; expression is cell cycle regulated, peaking in M/G1 and also subject to regulation by the cell integrity pathway
POB3		YML069	+	Subunit of the heterodimeric FACT complex (Spt16p-Pob3p), which facilitates RNA Pol II transcription elongation through nucleosomes by destabilizing and then reassembling nucleosome structure; interacts with DNA polymerase alpha (Pol1p)
POC4		YPL144	+	Chaperone component involved in 20S proteasome assembly; forms a heterodimer with Irc25p; green fluorescent protein (GFP)-fusion protein localizes to the cytoplasm; null mutant is viable, exhibits shortened telomeres
POX1	FOX1	YGL205	-	Fatty-acyl coenzyme A oxidase, involved in the fatty acid beta-oxidation pathway; localized to the peroxisomal matrix
PPR1		YLR014	+	Zinc finger transcription factor containing a Zn(2)-Cys(6) binuclear cluster domain, positively regulates transcription of genes involved in uracil biosynthesis; activity may be modulated by interaction with Tup1p

Gene Name	Alternate Gene Name	ORF	Up + or Down - regulation	Gene Description
PRM2		YIL037	-	Pheromone-regulated protein, predicted to have 4 transmembrane segments and a coiled coil domain; regulated by Ste12p
PRM6		YML047	-	Pheromone-regulated protein, predicted to have 2 transmembrane segments; regulated by Ste12p during mating
PRM9		YAR031	-	Pheromone-regulated protein with 3 predicted transmembrane segments and an FF sequence, a motif involved in COPII binding; member of DUP240 gene family
PRP16		YKR086	+	RNA helicase in the DEAH-box family involved in the second catalytic step of splicing, exhibits ATP-dependent RNA unwinding activity
PRP2	RNA2	YNR011	+	RNA-dependent ATPase in the DEAH-box family, required for activation of the spliceosome before the first transesterification step in RNA splicing
PRP9		YDL030	+	Subunit of the SF3a splicing factor complex, required for spliceosome assembly; acts after the formation of the U1 snRNP-pre-mRNA complex
PRR1		YKL115	+	Serine/threonine protein kinase that inhibits pheromone induced signalling downstream of MAPK, possibly at the level of the Ste12p transcription factor
PRS1	PRP1	YKL181	+	5-phospho-ribosyl-1(alpha)-pyrophosphate synthetase, involved in nucleotide, histidine, and tryptophan biosynthesis; one of five related enzymes, which are active as heteromultimeric complexes
PRY2	YFW12	YKR013	+	Protein of unknown function, has similarity to Pry1p and Pry3p and to the plant PR-1 class of pathogen related proteins
PSA1	MPG1|SRB 1|VIG9	YDL055	-	GDP-mannose pyrophosphorylase (mannose-1-phosphate guanyltransferase), synthesizes GDP-mannose from GTP and mannose-1-phosphate in cell wall biosynthesis; required for normal cell wall structure
PTA1	FUN39	YAL043	+	Subunit of holo-CPF, a multiprotein complex and functional homolog of mammalian CPSF, required for the cleavage and polyadenylation of mRNA and snoRNA 3' ends; involved in pre-tRNA processing; binds to the phosphorylated CTD of RNAPII
PTM1		YKL039	+	Protein of unknown function, copurifies with late Golgi vesicles containing the v-SNARE Tlg2p
PUF3		YLL013	-	Protein of the mitochondrial outer surface, links the Arp2/3 complex with the mitochore during anterograde mitochondrial movement; also binds to and promotes degradation of mRNAs for select nuclear-encoded mitochondrial proteins
PUG1		YER185	-	Plasma membrane protein involved in protoporphyrin uptake; similar in sequence to RSB1, which is involved in sphingoid long-chain base release
PUT3		YKL015	+	Transcriptional activator of proline utilization genes, constitutively binds PUT1 and PUT2 promoter sequences and undergoes a conformational change to form the active state; has a Zn(2)-Cys(6) binuclear cluster domain
PYC1		YGL062	-	Pyruvate carboxylase isoform, cytoplasmic enzyme that converts pyruvate to oxaloacetate; highly similar to isoform Pyc2p but differentially regulated; mutations in the human homolog are associated with lactic acidosis
PYK2		YOR347	-	Pyruvate kinase that appears to be modulated by phosphorylation; PYK2 transcription is repressed by glucose, and Pyk2p may be active under low glycolytic flux

Gene Name	Alternate Gene Name	ORF	Up + or Down - regulation	Gene Description
QDR1		YIL120	-	Multidrug transporter of the major facilitator superfamily, required for resistance to quinidine, ketoconazole, fluconazole, and barban
RAD59		YDL059	-	Protein involved in the repair of double-strand breaks in DNA during vegetative growth via recombination and single-strand annealing; anneals complementary single-stranded DNA; homologous to Rad52p
RDS1		YCR106	-	Zinc cluster transcription factor involved in conferring resistance to cycloheximide
REC104		YHR157	+	Protein involved in early stages of meiotic recombination; required for meiotic crossing over; forms a complex with Rec102p and Spo11p necessary during the initiation of recombination
RED1		YLR263	+	Protein component of the axial elements of the synaptonemal complex, involved in chromosome segregation during the first meiotic division; interacts with Hop1p; required for wild-type levels of Mek1p kinase activity
REG2		YBR050	-	Regulatory subunit of the Glc7p type-1 protein phosphatase; involved with Reg1p, Glc7p, and Snf1p in regulation of glucose-repressible genes, also involved in glucose-induced proteolysis of maltose permease
RER1		YCL001	-	Protein involved in retention of membrane proteins, including Sec12p, in the ER; localized to Golgi; functions as a retrieval receptor in returning membrane proteins to the ER
REV3	PSO1	YPL167	+	Catalytic subunit of DNA polymerase zeta, which is involved in DNA repair and translesion synthesis; required for mutagenesis induced by DNA damage
RGA1	DBM1|THE1	YOR127	-	GTPase-activating protein for the polarity-establishment protein Cdc42p; implicated in control of septin organization, pheromone response, and haploid invasive growth
RGT1		YKL038	+	Glucose-responsive transcription factor that regulates expression of several glucose transporter (HXT) genes in response to glucose; binds to promoters and acts both as a transcriptional activator and repressor
RHR2	GPP1	YIL053	-	Constitutively expressed isoform of DL-glycerol-3-phosphatase; involved in glycerol biosynthesis, induced in response to both anaerobic and, along with the Hor2p/Gpp2p isoform, osmotic stress
RIB2	PUS8	YOL066	-	DRAP deaminase, catalyzes the third step of the riboflavin biosynthesis pathway; cytoplasmic tRNA pseudouridine synthase involved in pseudouridylation of cytoplasmic tRNAs at position 32
RIB4		YOL143	+	Lumazine synthase (6,7-dimethyl-8-ribityllumazine synthase, also known as DMRL synthase); catalyzes synthesis of immediate precursor to riboflavin
RIT1		YMR283	-	2'-O-ribosyl phosphate transferase, modifies the initiator methionine tRNA at position 64 to distinguish it from elongator methionine tRNA
RNA14		YMR061	-	Cleavage and polyadenylation factor I (CF I) component involved in cleavage and polyadenylation of mRNA 3' ends; bridges interaction between Rna15p and Hrp1p in the CF I complex
ROD1		YOR018	+	Membrane protein; overexpression confers resistance to the GST substrate o-dinitrobenzene as well as to zinc and calcium; contains 2 PY motifs, which are required for Rod1p interaction with Rsp5p, a hect-type ubiquitin ligase
RPB11		YOL005	+	RNA polymerase II subunit B12.5; part of central core; similar to Rpc19p and bacterial alpha subunit
RPB3		YIL021	-	RNA polymerase II third largest subunit B44, part of central core; similar to prokaryotic alpha subunit
RPC37		YKR025	+	RNA polymerase III subunit C37

Gene Name	Alternate Gene Name	ORF	Up + or Down - regulation	Gene Description
RPE1	EPI1|POS18	YJL121	-	D-ribulose-5-phosphate 3-epimerase, catalyzes a reaction in the non-oxidative part of the pentose-phosphate pathway; mutants are sensitive to oxidative stress
RPL14B		YHL001	+	Protein component of the large (60S) ribosomal subunit, nearly identical to Rpl14Ap and has similarity to rat L14 ribosomal protein
RPL17A	RPL17	YKL180	+	Protein component of the large (60S) ribosomal subunit, nearly identical to Rpl17Bp and has similarity to E. coli L22 and rat L17 ribosomal proteins; copurifies with the Dam1 complex (aka DASH complex)
RPL17B		YJL177	+	Protein component of the large (60S) ribosomal subunit, nearly identical to Rpl17Ap and has similarity to E. coli L22 and rat L17 ribosomal proteins
RPL1A	SSM1	YPL220	+	N-terminally acetylated protein component of the large (60S) ribosomal subunit, nearly identical to Rpl1Bp and has similarity to E. coli L1 and rat L10a ribosomal proteins; rpl1a rpl1b double null mutation is lethal
RPL1B	SSM2	YGL135	+	N-terminally acetylated protein component of the large (60S) ribosomal subunit, nearly identical to Rpl1Ap and has similarity to E. coli L1 and rat L10a ribosomal proteins; rpl1a rpl1b double null mutation is lethal
RPL37A		YLR185	+	Protein component of the large (60S) ribosomal subunit, has similarity to Rpl37Bp and to rat L37 ribosomal protein
RPL37B		YDR500	+	Protein component of the large (60S) ribosomal subunit, has similarity to Rpl37Ap and to rat L37 ribosomal protein
RPL39	PUB2|RPL46|SPB2	YJL189	+	Protein component of the large (60S) ribosomal subunit, has similarity to rat L39 ribosomal protein; required for ribosome biogenesis; exhibits genetic interactions with SIS1 and PAB1
RPL40A	CEP52A|UB11|UBI1	YIL148	+	Fusion protein, identical to Rpl40Bp, that is cleaved to yield ubiquitin and a ribosomal protein of the large (60S) ribosomal subunit with similarity to rat L40; ubiquitin may facilitate assembly of the ribosomal protein into ribosomes
RPO21	RPB1|RPB220|SUA8	YDL140	-	RNA polymerase II largest subunit B220, part of central core; phosphorylation of C-terminal heptapeptide repeat domain regulates association with transcription and splicing factors; similar to bacterial beta-prime
RPP1A	RPLA1	YDL081	+	Ribosomal stalk protein P1 alpha, involved in the interaction between translational elongation factors and the ribosome; accumulation of P1 in the cytoplasm is regulated by phosphorylation and interaction with the P2 stalk component
RPS12		YOR369	+	Protein component of the small (40S) ribosomal subunit; has similarity to rat ribosomal protein S12
RPS2	RPS4|SUP138|SUP38|SUP44	YGL123	+	Protein component of the small (40S) subunit, essential for control of translational accuracy; has similarity to E. coli S5 and rat S2 ribosomal proteins
RPS21A	RPS25	YKR057	+	Protein component of the small (40S) ribosomal subunit; nearly identical to Rps21Bp and has similarity to rat S21 ribosomal protein
RPS21B		YJL136	+	Protein component of the small (40S) ribosomal subunit; nearly identical to Rps21Ap and has similarity to rat S21 ribosomal protein

Gene Name	Alternate Gene Name	ORF	Up + or Down - regulation	Gene Description
RPS25A	RPS31A	YGR027	-	Protein component of the small (40S) ribosomal subunit; nearly identical to Rps25Bp and has similarity to rat S25 ribosomal protein
RPS26A	RPS26	YGL189	+	Protein component of the small (40S) ribosomal subunit; nearly identical to Rps26Bp and has similarity to rat S26 ribosomal protein
RPS28A	RPS33A	YOR167	+	Protein component of the small (40S) ribosomal subunit; nearly identical to Rps28Bp and has similarity to rat S28 ribosomal protein
RPS28B	RPS33B	YLR264	+	Protein component of the small (40S) ribosomal subunit; nearly identical to Rps28Ap and has similarity to rat S28 ribosomal protein
RPS30A		YLR287	+	Protein component of the small (40S) ribosomal subunit; nearly identical to Rps30Bp and has similarity to rat S30 ribosomal protein
RPS31	RPS37|UBI 3	YLR167	+	Fusion protein that is cleaved to yield a ribosomal protein of the small (40S) subunit and ubiquitin; ubiquitin may facilitate assembly of the ribosomal protein into ribosomes; interacts genetically with translation factor eIF2B
RRF1	FIL1|KIM4	YHR038	-	Mitochondrial ribosome recycling factor, essential for mitochondrial protein synthesis and for the maintenance of the respiratory function of mitochondria
RRN3		YKL125	+	Protein required for transcription of rDNA by RNA polymerase I; transcription factor independent of DNA template; involved in recruitment of RNA polymerase I to rDNA
RRP6		YOR001	-	Exonuclease component of the nuclear exosome; contributes to the quality-control system that retains and degrades aberrant mRNAs in the nucleus
RRP9		YPR137	-	Protein involved in pre-rRNA processing, associated with U3 snRNP; component of small ribosomal subunit (SSU) processosome; ortholog of the human U3-55k protein
RTA1		YGR213	+	Protein involved in 7-aminocholesterol resistance; has seven potential membrane-spanning regions
RTS2	YOR29-28	YOR077	-	Basic zinc-finger protein, similar to human and mouse Kin17 proteins which are chromatin-associated proteins involved in UV response and DNA replication
RTS3		YGR161	-	Putative component of the protein phosphatase type 2A complex
RTS3		YGR161	-	Putative component of the protein phosphatase type 2A complex
SAG1	AG(ALPH A)1	YJR004	-	Alpha-agglutinin of alpha-cells, binds to Aga1p during agglutination, N-terminal half is homologous to the immunoglobulin superfamily and contains binding site for a-agglutinin, C-terminal half is highly glycosylated and contains GPI anchor
SAM4		YPL273	+	S-adenosylmethionine-homocysteine methyltransferase, functions along with Mht1p in the conversion of S-adenosylmethionine (AdoMet) to methionine to control the methionine/AdoMet ratio
SAS4		YDR181	+	Subunit of the SAS complex (Sas2p, Sas4p, Sas5p), which acetylates free histones and nucleosomes and regulates transcriptional silencing; required for the HAT activity of Sas2p
SAS5		YOR213	-	Subunit of the SAS complex (Sas2p, Sas4p, Sas5p), which acetylates free histones and nucleosomes and regulates transcriptional silencing; stimulates Sas2p HAT activity
SBA1		YKL116	+	Co-chaperone that binds to and regulates Hsp90 family chaperones; important for pp60v-src activity in yeast; homologous to the mammalian p23 proteins and like p23 can regulate telomerase activity

Gene Name	Alternate Gene Name	ORF	Up + or Down - regulation	Gene Description
SBE2		YDR351	-	Protein involved in the transport of cell wall components from the Golgi to the cell surface; required for bud growth
SCM3		YDL139	-	Nonhistone component of centromeric chromatin that binds stoichiometrically to CenH3-H4 histones, required for kinetochore assembly; contains nuclear export signal (NES); required for G2/M progression and localization of Cse4p
SDS22	EGP1	YKL193	+	Conserved nuclear regulatory subunit of Glc7p type 1 protein serine-threonine phosphatase (PP1), functions positively with Glc7p to promote dephosphorylation of nuclear substrates required for chromosome transmission during mitosis
SDS3		YIL084	-	Component of the Rpd3p/Sin3p deacetylase complex required for its structural integrity and catalytic activity, involved in transcriptional silencing and required for sporulation; cells defective in SDS3 display pleiotropic phenotypes
SEC53	ALG4	YFL045	+	Phosphomannomutase, involved in synthesis of GDP-mannose and dolichol-phosphate-mannose; required for folding and glycosylation of secretory proteins in the ER lumen
SED1		YDR077	+	Major stress-induced structural GPI-cell wall glycoprotein in stationary-phase cells, associates with translating ribosomes, possible role in mitochondrial genome maintenance; ORF contains two distinct variable minisatellites
SEN2		YLR105	+	Subunit of the tRNA splicing endonuclease, which is composed of Sen2p, Sen15p, Sen34p, and Sen54p; Sen2p contains the active site for tRNA 5' splice site cleavage and has similarity to Sen34p and to Archaeal tRNA splicing endonuclease
SEO1		YAL067	-	Putative permease, member of the allantoate transporter subfamily of the major facilitator superfamily; mutation confers resistance to ethionine sulfoxide
SER3		YER081	+	3-phosphoglycerate dehydrogenase, catalyzes the first step in serine and glycine biosynthesis; isozyme of Ser33p
SFA1	ADH5	YDL168	+	Bifunctional enzyme containing both alcohol dehydrogenase and glutathione-dependent formaldehyde dehydrogenase activities, functions in formaldehyde detoxification and formation of long chain and complex alcohols, regulated by Hog1p-Sko1p
SFK1		YKL051	+	Plasma membrane protein that may act together with or upstream of Stt4p to generate normal levels of the essential phospholipid PI4P, at least partially mediates proper localization of Stt4p to the plasma membrane
SHE2		YKL130	+	RNA-binding protein that binds specific mRNAs and interacts with She3p; part of the mRNA localization machinery that restricts accumulation of certain proteins to the bud
SHE9	MDM33	YDR393	-	Mitochondrial inner membrane protein required for normal mitochondrial morphology, may be involved in fission of the inner membrane; forms a homo-oligomeric complex
SHG1	CPS15	YBR258	-	Subunit of the COMPASS (Set1C) complex, which methylates histone H3 on lysine 4 and is required in transcriptional silencing near telomeres
SHU2		YDR078	+	Protein of unassigned function involved in mutation suppression, important for error-free repair of spontaneous and induced DNA lesions to protect the genome from mutation; associates with Shu1p, Psy3p, and Csm2p

Gene Name	Alternate Gene Name	ORF	Up + or Down - regulation	Gene Description
SIP18		YMR175	+	Protein of unknown function whose expression is induced by osmotic stress
SKG1	YKR099C-A	YKR100	+	Transmembrane protein with a role in cell wall polymer composition; localizes on the inner surface of the plasma membrane at the bud and in the daughter cell
SMC5		YOL034	+	Structural maintenance of chromosomes (SMC) protein; essential subunit of the Mms21-Smc5-Smc6 complex; required for growth and DNA repair; S. pombe homolog forms a heterodimer with S. pombe Rad18p that is involved in DNA repair
SMY1		YKL079	+	Protein that interacts with Myo2p, proposed to be involved in exocytosis; N-terminal domain is related to the motor domain of kinesins
SNF6		YHL025	-	Subunit of the SWI/SNF chromatin remodeling complex involved in transcriptional regulation; functions interdependently in transcriptional activation with Snf2p and Snf5p
SNO2		YNL334	-	Protein of unknown function, nearly identical to Sno3p; expression is induced before the diauxic shift and also in the absence of thiamin
SNO3		YFL060	-	Protein of unknown function, nearly identical to Sno2p; expression is induced before the diauxic shift and also in the absence of thiamin
SNO4	HSP34	YMR322	-	Possible chaperone and cysteine protease with similarity to E. coli Hsp31 and S. cerevisiae Hsp31p, Hsp32p, and Hsp33p; member of the DJ-1/ThiJ/PfpI superfamily; may have a role in pyridoxine metabolism
SNU114	GIN10	YKL173	+	GTPase component of U5 snRNP involved in mRNA splicing via spliceosome; binds directly to U5 snRNA; proposed to be involved in conformational changes of the spliceosome; similarity to ribosomal translocation factor EF-2
SNU13		YEL026	+	RNA binding protein, part of U3 snoRNP involved in rRNA processing, part of U4/U6-U5 tri-snRNP involved in mRNA splicing, similar to human 15.5K protein
SNZ2		YNL333	-	Member of a stationary phase-induced gene family; transcription of SNZ2 is induced prior to diauxic shift, and also in the absence of thiamin in a Thi2p-dependent manner; forms a coregulated gene pair with SNO2; interacts with Thi11p
SNZ3		YFL059	-	Member of a stationary phase-induced gene family; transcription of SNZ2 is induced prior to diauxic shift, and also in the absence of thiamin in a Thi2p-dependent manner; forms a coregulated gene pair with SNO3
SPB4		YFL002	-	Putative ATP-dependent RNA helicase, nucleolar protein required for synthesis of 60S ribosomal subunits at a late step in the pathway; sediments with 66S pre-ribosomes in sucrose gradients
SPL2		YHR136	-	Protein with similarity to cyclin-dependent kinase inhibitors, overproduction suppresses a plc1 null mutation; green fluorescent protein (GFP)-fusion protein localizes to the cytoplasm in a punctate pattern
SPO11		YHL022	-	Meiosis-specific protein that initiates meiotic recombination by catalyzing the formation of double-strand breaks in DNA via a transesterification reaction; required for homologous chromosome pairing and synaptonemal complex formation
SPO75		YLL005	-	Meiosis-specific protein of unknown function, required for spore wall formation during sporulation; dispensable for both nuclear divisions during meiosis

Gene Name	Alternate Gene Name	ORF	Up + or Down - regulation	Gene Description
SPS19	SPX19	YNL202	-	Peroxisomal 2,4-dienoyl-CoA reductase, auxiliary enzyme of fatty acid beta-oxidation; homodimeric enzyme required for growth and sporulation on petroselineate medium; expression induced during late sporulation and in the presence of oleate
SPS22		YCL048	-	Protein of unknown function, redundant with Sps2p for the organization of the beta-glucan layer of the spore wall
SPS4		YOR313	-	Protein whose expression is induced during sporulation; not required for sporulation; heterologous expression in E. coli induces the SOS response that senses DNA damage
SPT23		YKL020	+	ER membrane protein involved in regulation of OLE1 transcription, acts with homolog Mga2p; inactive ER form dimerizes and one subunit is then activated by ubiquitin/proteasome-dependent processing followed by nuclear targeting
SRB4	MED17	YER022	-	Subunit of the RNA polymerase II mediator complex; associates with core polymerase subunits to form the RNA polymerase II holoenzyme; essential for transcriptional regulation
SRD1		YCR018	-	Protein involved in the processing of pre-rRNA to mature rRNA; contains a C2/C2 zinc finger motif; srd1 mutation suppresses defects caused by the rrp1-1 mutation
SRL3		YKR091	+	Cytoplasmic protein that, when overexpressed, suppresses the lethality of a rad53 null mutation; potential Cdc28p substrate
SRM1	MTR1|PRP 20	YGL097	-	Nucleotide exchange factor for Gsp1p, localizes to the nucleus, required for nucleocytoplasmic trafficking of macromolecules; suppressor of the pheromone response pathway; potentially phosphorylated by Cdc28p
SRP21		YKL122	+	Subunit of the signal recognition particle (SRP), which functions in protein targeting to the endoplasmic reticulum membrane; not found in mammalian SRP; forms a pre-SRP structure in the nucleolus that is translocated to the cytoplasm
SRT1		YMR101	-	Cis-prenyltransferase involved in synthesis of long-chain dolichols (19-22 isoprene units; as opposed to Rer2p which synthesizes shorter-chain dolichols); localizes to lipid bodies; transcription is induced during stationary phase
SRX1		YKL086	+	Sulfiredoxin, contributes to oxidative stress resistance by reducing cysteine-sulfinic acid groups in the peroxiredoxins Tsa1p and Ahp1p that are formed upon exposure to oxidants; conserved in higher eukaryotes
SSA3	YG106	YBL075	+	ATPase involved in protein folding and the response to stress; plays a role in SRP-dependent cotranslational protein-membrane targeting and translocation; member of the heat shock protein 70 (HSP70) family; localized to the cytoplasm
SSH4	MLF4	YKL124	+	Vacuolar protein that presumably functions within the endosomal-vacuolar trafficking pathway, affecting events that determine whether plasma membrane proteins are degraded or routed to the plasma membrane
STE24	AFC1|PIO2	YJR117	+	Highly conserved zinc metalloprotease that functions in two steps of a-factor maturation, C-terminal CAAX proteolysis and the first step of N-terminal proteolytic processing; contains multiple transmembrane spans

Gene Name	Alternate Gene Name	ORF	Up + or Down - regulation	Gene Description
STE50		YCL032	-	Protein involved in mating response, invasive/filamentous growth, and osmotolerance, acts as an adaptor that links G protein-associated Cdc42p-Ste20p complex to the effector Ste11p to modulate signal transduction
STL1		YDR536	+	Glycerol proton symporter of the plasma membrane, subject to glucose-induced inactivation, strongly but transiently induced when cells are subjected to osmotic shock
SUC2		YIL162	-	Invertase, sucrose hydrolyzing enzyme; a secreted, glycosylated form is regulated by glucose repression, and an intracellular, nonglycosylated enzyme is produced constitutively
SUI1	MOF2|RFR 1	YNL244	+	Translation initiation factor eIF1; component of a complex involved in recognition of the initiator codon; modulates translation accuracy at the initiation phase
SWD2	CPS35|SAF 37	YKL018	+	Subunit of the COMPASS (Set1C) complex, which methylates histone H3 on lys 4 and is involved in telomeric silencing; subunit of CPF (cleavage and polyadenylation factor), a complex involved in RNAP II transcription termination
SWF1	PSL10	YDR126	-	Palmitoyltransferase that acts on the SNAREs Snc1p, Syn8p, Tlg1p and likely on all SNAREs; member of a family of putative palmitoyltransferases containing an Asp-His-His-Cys-cysteine rich (DHHC-CRD) domain; may have a role in vacuole fusion
SWH1	OSH1|YAR 044W	YAR042	-	Protein similar to mammalian oxysterol-binding protein; contains ankyrin repeats; localizes to the Golgi and the nucleus-vacuole junction
SYF2	NTC31	YGR129	-	Component of the spliceosome complex involved in pre-mRNA splicing; involved in regulation of cell cycle progression
SYG1		YIL047	-	Plasma membrane protein of unknown function; truncation and overexpression suppresses lethality of G-alpha protein deficiency
TAT1	TAP1|VAP1	YBR069	+	Amino acid transport protein for valine, leucine, isoleucine, and tyrosine, low-affinity tryptophan and histidine transporter; overexpression confers FK506 and FTY720 resistance
TAT2	LTG3|SAB2 |SCM2|TAP 2	YOL020	-	High affinity tryptophan and tyrosine permease, overexpression confers FK506 and FTY720 resistance
TAZ1		YPR140	-	Lyso-phosphatidylcholine acyltransferase, required for normal phospholipid content of mitochondrial membranes; may remodel acyl groups of cardiolipin in the inner membrane; similar to human tafazzin, which is implicated in Barth syndrome
TCB3		YML072	-	Lipid-binding protein, localized to the bud via specific mRNA transport; non-tagged protein detected in a phosphorylated state in mitochondria; GFP-fusion protein localizes to the cell periphery; C-termini of Tcb1p, Tcb2p and Tcb3p interact
TEF1	EF-1 alpha	YPR080	+	Translational elongation factor EF-1 alpha; also encoded by TEF2; functions in the binding reaction of aminoacyl-tRNA (AA-tRNA) to ribosomes
TEF4	EFC1	YKL081	+	Translation elongation factor EF-1 gamma
TEM1		YML064	+	GTP-binding protein of the ras superfamily involved in termination of M-phase; controls actomyosin and septin dynamics during cytokinesis
TEX1		YNL253	-	Protein involved in mRNA export, component of the transcription export (TREX) complex

Gene Name	Alternate Gene Name	ORF	Up + or Down - regulation	Gene Description
TFA2		YKR062	+	TFIIE small subunit, involved in RNA polymerase II transcription initiation
TFB3	RIG2	YDR460	-	Subunit of TFIIH and nucleotide excision repair factor 3 complexes, involved in transcription initiation, required for nucleotide excision repair; ring finger protein similar to mammalian CAK and TFIIH subunit
TFC3	FUN24|TSV115	YAL001	-	Largest of six subunits of the RNA polymerase III transcription initiation factor complex (TFIIIC); part of the TauB domain of TFIIIC that binds DNA at the BoxB promoter sites of tRNA and similar genes; cooperates with Tfc6p in DNA binding
TGL2		YDR058	-	Protein with lipolytic activity towards triacylglycerols and diacylglycerols when expressed in E. coli; role in yeast lipid degradation is unclear
TGL4	STC1	YKR089	+	Triacylglycerol lipase involved in triacylglycerol mobilization and degradation; found in lipid particles; potential Cdc28p substrate
THI12		YNL332	-	Protein involved in synthesis of the thiamine precursor hydroxymethylpyrimidine (HMP); member of a subtelomeric gene family including THI5, THI11, THI12, and THI13
THI13		YDL244	-	Protein involved in synthesis of the thiamine precursor hydroxymethylpyrimidine (HMP); member of a subtelomeric gene family including THI5, THI11, THI12, and THI13
THI21		YPL258	-	Hydroxymethylpyrimidine phosphate kinase, involved in the last steps in thiamine biosynthesis; member of a gene family with THI20 and THI22; functionally redundant with Thi20p
THI4	ESP35|MOL1	YGR144	-	Thiazole synthase, catalyzes formation of the thiazole moiety of thiamin pyrophosphate; required for thiamine biosynthesis and for mitochondrial genome stability
THI5		YFL058	-	Protein involved in synthesis of the thiamine precursor hydroxymethylpyrimidine (HMP); member of a subtelomeric gene family including THI5, THI11, THI12, and THI13
THI7	THI10	YLR237	+	Plasma membrane transporter responsible for the uptake of thiamine, member of the major facilitator superfamily of transporters; mutation of human ortholog causes thiamine-responsive megaloblastic anemia
THI71	YOR29-22	YOR071	-	Transporter of thiamine or related compound; shares sequence similarity to Thi7p; proposed to be involved in 5-fluorocytosine sensitivity
THI72		YOR192	-	Transporter of thiamine or related compound; shares sequence similarity with Thi7p
THR1		YHR025	+	Homoserine kinase, conserved protein required for threonine biosynthesis; expression is regulated by the GCN4-mediated general amino acid control pathway
TIM22		YDL217	+	Component of the mitochondrial Tim54p-Tim22p complex involved in insertion of polytopic proteins into the inner membrane
TIP1		YBR067	+	Major cell wall mannoprotein with possible lipase activity; transcription is induced by heat- and cold-shock; member of the Srp1p/Tip1p family of serine-alanine-rich proteins
TIS11	CTH2	YLR136	-	mRNA-binding protein expressed during iron starvation; binds to a sequence element in the 3'-untranslated regions of specific mRNAs to mediate their degradation; involved in iron homeostasis
TMA19	MMI1|RBF18	YKL056	+	Protein that associates with ribosomes; homolog of translationally controlled tumor protein; green fluorescent protein (GFP)-fusion protein localizes to the cytoplasm and relocates to the mitochondrial outer surface upon oxidative stress

Gene Name	Alternate Gene Name	ORF	Up + or Down - regulation	Gene Description
TMA20	RBF20	YER007	-	Protein of unknown function that associates with ribosomes and has a putative RNA binding domain; interacts with Tma22p; null mutant exhibits translation defects; has homology to human oncogene MCT-1
TPO5		YKL174	+	Protein involved in excretion of putrescine and spermidine; putative polyamine transporter in the Golgi or post-Golgi vesicles
TPT1		YOL102	-	tRNA 2'-phosphotransferase, catalyzes the final step in yeast tRNA splicing: the transfer of the 2'-PO(4) from the splice junction to NAD(+) to form ADP-ribose 1"-2"cyclic phosphate and nicotinamide
TRK2	RPD2	YKR050	+	Component of the Trk1p-Trk2p potassium transport system
TRS31		YDR472	+	One of 10 subunits of the transport protein particle (TRAPP) complex of the cis-Golgi which mediates vesicle docking and fusion; involved in endoplasmic reticulum (ER) to Golgi membrane traffic
TRX3		YCR083	+	Mitochondrial thioredoxin, highly conserved oxidoreductase required to maintain the redox homeostasis of the cell, forms the mitochondrial thioredoxin system with Trr2p, redox state is maintained by both Trr2p and Glr1p
TUL1		YKL034	+	Golgi-localized RING-finger ubiquitin ligase (E3), involved in ubiquitinating and sorting membrane proteins that contain polar transmembrane domains to multivesicular bodies for delivery to the vacuole for quality control purposes
TVP38		YKR088	+	Integral membrane protein localized to late Golgi vesicles along with the v-SNARE Tlg2p; green fluorescent protein (GFP)-fusion protein localizes to the cytoplasm in a punctate pattern
TYE7	SGC1	YOR344	-	Serine-rich protein that contains a basic-helix-loop-helix (bHLH) DNA binding motif; binds E-boxes of glycolytic genes and contributes to their activation; may function as a transcriptional activator in Ty1-mediated gene expression
UBA1		YKL210	+	Ubiquitin activating enzyme (E1), involved in ubiquitin-mediated protein degradation and essential for viability
UBC11		YOR339	-	Ubiquitin-conjugating enzyme most similar in sequence to Xenopus ubiquitin-conjugating enzyme E2-C, but not a true functional homolog of this E2; unlike E2-C, not required for the degradation of mitotic cyclin Clb2
UBX6	CUI2	YJL048	+	UBX (ubiquitin regulatory X) domain-containing protein that interacts with Cdc48p, transcription is repressed when cells are grown in media containing inositol and choline
URA1		YKL216	+	Dihydroorotate dehydrogenase, catalyzes the fourth enzymatic step in the de novo biosynthesis of pyrimidines, converting dihydroorotic acid into orotic acid
URA3		YEL021	-	Orotidine-5'-phosphate (OMP) decarboxylase, catalyzes the sixth enzymatic step in the de novo biosynthesis of pyrimidines, converting OMP into uridine monophosphate (UMP); converts 5-FOA into 5-fluorouracil, a toxic compound
URB1	NPA1	YKL014	-	Nucleolar protein required for the normal accumulation of 25S and 5.8S rRNAs, associated with the 27SA2 pre-ribosomal particle; proposed to be involved in the biogenesis of the 60S ribosomal subunit
URH1		YDR400	-	Uridine nucleosidase (uridine-cytidine N-ribohydrolase), cleaves N-glycosidic bonds in nucleosides; involved in the pyrimidine salvage and nicotinamide riboside salvage pathways

Gene Name	Alternate Gene Name	ORF	Up + or Down - regulation	Gene Description
URM1		YIL013	+	Ubiquitin-like protein with weak sequence similarity to ubiquitin; depends on the E1-like activating enzyme Uba4p; molecular function of the Urm1p pathway is unknown, but it is required for normal growth, particularly at high temperature
USE1	SLT1	YGL098	+	Essential SNARE protein localized to the ER, involved in retrograde traffic from the Golgi to the ER; forms a complex with the SNAREs Sec22p, Sec20p and Ufe1p
UTH1		YKR042	+	Mitochondrial outer membrane and cell wall localized SUN family member required for mitochondrial autophagy; involved in the oxidative stress response, life span during starvation, mitochondrial biogenesis, and cell death
UTP11		YKL099	+	Nucleolar protein, component of the small subunit (SSU) processome containing the U3 snoRNA that is involved in processing of pre-18S rRNA
UTP30		YKR060	+	Possible U3 snoRNP protein involved in maturation of pre-18S rRNA, based on computational analysis of large-scale protein-protein interaction data
VAC7		YNL054	-	Integral vacuolar membrane protein involved in vacuole inheritance and morphology; activates Fab1p kinase activity under basal conditions and also after hyperosmotic shock
VAN1	LDB13|VRG7|VRG8	YML115	-	Component of the mannan polymerase I, which contains Van1p and Mnn9p and is involved in the first steps of mannan synthesis; mutants are vanadate-resistant
VAR1	VARI	Q0140	-	Mitochondrial ribosomal protein of the small subunit, mitochondrially-encoded; polymorphic in different strains due to variation in number of AAT (asparagine) codons; translated near the mitochondrial inner membrane
VIP1		YLR410	-	Inositol hexakisphosphate (IP6) and inositol heptakisphosphate (IP7) kinase; generation of IP7 by Vip1p is important for phosphate signaling; likely involved in cortical actin cytoskeleton function, by analogy with S. pombe ortholog asp1
VPH2	CLS10|VMA12	YKL117	+	Integral membrane protein required for vacuolar H+-ATPase (V-ATPase) function, although not an actual component of the V-ATPase complex; functions in the assembly of the V-ATPase; localized to the endoplasmic reticulum (ER)
XRS2		YDR369	+	Protein required for DNA repair; component of the Mre11 complex, which is involved in double strand breaks, meiotic recombination, telomere maintenance, and checkpoint signaling
XYL2		YLR070	-	Xylitol dehydrogenase, converts xylitol to D-xylulose in the endogenous xylose utilization pathway
YAP3		YHL009	-	Basic leucine zipper (bZIP) transcription factor
YAT1		YAR035	-	Outer mitochondrial carnitine acetyltransferase, minor ethanol-inducible enzyme involved in transport of activated acyl groups from the cytoplasm into the mitochondrial matrix; phosphorylated
YAT2		YER024	-	Carnitine acetyltransferase; has similarity to Yat1p, which is a carnitine acetyltransferase associated with the mitochondrial outer membrane
YCT1		YLL055	-	High-affinity cysteine-specific transporter with similarity to the Dal5p family of transporters; green fluorescent protein (GFP)-fusion protein localizes to the endoplasmic reticulum; YCT1 is not an essential gene

Gene Name	Alternate Gene Name	ORF	Up + or Down - regulation	Gene Description
YEF3	TEF3	YLR249	-	Translational elongation factor 3, stimulates the binding of aminoacyl-tRNA (AA-tRNA) to ribosomes by releasing EF-1 alpha from the ribosomal complex; contains two ABC cassettes; binds and hydrolyses ATP
YET1		YKL065	+	Endoplasmic reticulum transmembrane protein; may interact with ribosomes, based on co-purification experiments; homolog of human BAP31 protein
YKT6		YKL196	+	Vesicle membrane protein (v-SNARE) with acyltransferase activity; involved in trafficking to and within the Golgi, endocytic trafficking to the vacuole, and vacuolar fusion; membrane localization due to prenylation at the carboxy-terminus
YKU80	HDF2	YMR106	-	Subunit of the telomeric Ku complex (Yku70p-Yku80p), involved in telomere length maintenance, structure and telomere position effect; relocates to sites of double-strand cleavage to promote nonhomologous end joining during DSB repair
YPS5		YGL259	-	Protein with similarity to GPI-anchored aspartic proteases such as Yap1p and Yap3p
YPT52		YKR014	+	GTPase, similar to Ypt51p and Ypt53p and to mammalian rab5; required for vacuolar protein sorting and endocytosis
YRA2		YKL214	+	Member of the REF (RNA and export factor binding proteins) family; when overexpressed, can substitute for the function of Yra1p in export of poly(A)+ mRNA from the nucleus
YRF1-1	YRF1	YDR545	-	Helicase encoded by the Y' element of subtelomeric regions, highly expressed in the mutants lacking the telomerase component TLC1; potentially phosphorylated by Cdc28p
YRF1-2	YRF1	YER190	-	Helicase encoded by the Y' element of subtelomeric regions, highly expressed in the mutants lacking the telomerase component TLC1; potentially phosphorylated by Cdc28p
YRF1-3	YRF1	YGR296	-	Helicase encoded by the Y' element of subtelomeric regions, highly expressed in the mutants lacking the telomerase component TLC1; potentially phosphorylated by Cdc28p
YRF1-4	YRF1	YLR466	-	Helicase encoded by the Y' element of subtelomeric regions, highly expressed in the mutants lacking the telomerase component TLC1; potentially phosphorylated by Cdc28p
YRF1-5	YRF1	YLR467	-	Helicase encoded by the Y' element of subtelomeric regions, highly expressed in the mutants lacking the telomerase component TLC1; potentially phosphorylated by Cdc28p
YRF1-6	YRF1	YNL339	-	Helicase encoded by the Y' element of subtelomeric regions, highly expressed in the mutants lacking the telomerase component TLC1; potentially phosphorylated by Cdc28p
YRF1-7	YRF1	YPL283	-	Helicase encoded by the Y' element of subtelomeric regions, highly expressed in the mutants lacking the telomerase component TLC1; potentially phosphorylated by Cdc28p
YRO2		YBR054	-	Putative protein of unknown function; the authentic, non-tagged protein is detected in a phosphorylated state in highly purified mitochondria in high-throughput studies; transcriptionally regulated by Haa1p
YSP3		YOR003	-	Putative precursor to the subtilisin-like protease III
YSR3	LBP2	YKR053	+	Dihydrosphingosine 1-phosphate phosphatase, membrane protein involved in sphingolipid metabolism; has similarity to Lcb3p
YTM1		YOR272	+	Constituent of 66S pre-ribosomal particles, required for maturation of the large ribosomal subunit
ZRG17		YNR039	-	Endoplasmic reticulum protein of unknown function, transcription is induced under conditions of zinc deficiency; mutant phenotype suggests a role in uptake of zinc

Gene Name	Alternate Gene Name	ORF	Up + or Down - regulation	Gene Description
ZRT3		YKL175	+	Vacuolar membrane zinc transporter, transports zinc from storage in the vacuole to the cytoplasm when needed; transcription is induced under conditions of zinc deficiency
